# Safety and Efficacy of the East Coast Fever Muguga Cocktail Vaccine: A Systematic Review

**DOI:** 10.3390/vaccines9111318

**Published:** 2021-11-12

**Authors:** Fiona K. Allan, Andrew R. Peters

**Affiliations:** Centre for Supporting Evidence Based Interventions-Livestock, The Royal (Dick) School of Veterinary Studies, The University of Edinburgh, Midlothian EH25 9RG, UK; fionakallan@gmail.com

**Keywords:** East Coast fever, cattle, Muguga cocktail, vaccine, safety, efficacy

## Abstract

Immunisation of livestock with high quality vaccines is considered an essential approach to controlling many animal diseases. The only currently available commercial vaccine to protect cattle from East Coast fever (ECF), a tick-borne disease caused by *Theileria parva*, is an unconventional “infection and treatment method” (ITM) involving administration of a combination of live *T. parva* isolates, referred to as the “Muguga cocktail”, and simultaneous treatment with long-acting oxytetracycline. Veterinary vaccine research and development typically involves studies designed to demonstrate vaccine quality, safety, and efficacy; however, as there were no such purpose-designed registration studies conducted for the Muguga cocktail, evidence for safety and efficacy is solely based on that which is available in the clinical literature. An extensive systematic review was conducted to analyse the evidence available in the literature in order to establish the safety and efficacy of the Muguga cocktail vaccine. A combination of meta-analyses and narrative summaries was conducted. A total of 61 studies met the criteria to be included in the systematic review. The majority of studies demonstrated or reported in favour of the vaccine with regards to safety and efficacy of the Muguga cocktail vaccine. Proximity to buffalo often resulted in reduced vaccine efficacy, and reports of shed and transmission of vaccine components affected the overall interpretation of safety. Better understanding of control options for this devastating livestock disease is important for policymakers and livestock keepers, enabling them to make informed decisions with regards to the health of their animals and their livelihoods.

## 1. Introduction

Pastoralist peoples in Africa inhabit some of the harshest environments in the world where livestock provide both health and socioeconomic wellbeing to these communities [1,2]. In particular, cattle have diverse roles and make vital contributions to livelihoods and economies in Africa; therefore, the ever-present threat of an often fatal, endemic, bovine disease is a hindrance to this livestock resource, with requirement for sustainable preventive solutions [3]. 

East Coast fever (ECF), caused by the apicomplexan parasite *Theileria parva* [4] and transmitted by the brown ear tick *Rhipicephalus appendiculatus,* is a fatal disease of cattle in eastern and southern Africa (see recent review by Nene et al. [5]). Around 50 million cattle are at risk (including ten million calves annually), and mortality rates for untreated ECF can reach 100% in nonendemic areas. The global economic impact on the livestock industry is estimated at USD 596 million annually [6]. Control has relied mostly on regular acaricide treatment [7] and antitheilerial drugs [8], but no effective conventional vaccine has yet been developed, possibly due to an incomplete understanding of the necessary immune mechanisms and how to stimulate them [5,9]. An atypical vaccination procedure, known as the “infection and treatment method” (ITM), was developed in the 1970s [10,11,12]. This relies on infection of animals with a potentially lethal dose of live *T. parva* sporozoites with concurrent treatment of long-acting oxytetracycline to control clinical symptoms. 

The subject of the current review is an ITM formulation based on three stocks of *T. parva*: Muguga, Kiambu 5, and Serengeti transformed [12,13]. It had become evident during development of the ITM that immunisation with one isolate of the parasite did not necessarily confer immunity to challenge with other known isolates. However, an ITM formulation based on three different stocks of parasite conferred immunity to challenge with several heterologous stocks [12]. The three stocks (Muguga, Kiambu 5, and Serengeti transformed) are known collectively as the Muguga cocktail, which has been used to immunise cattle across broad areas of East Africa. 

Although ECF-ITM has been available for almost four decades, its deployment has been inconsistent due to various reasons. First, the complexity of manufacturing the vaccine stabilate raised doubts as to whether consistent, commercial-scale batches could be produced. Secondly, there were epidemiological concerns that the Muguga cocktail would not induce protection against field strains found in all geographical situations. Third, as immunisation with live parasites can result in persistently infected or carrier animals, the possibility exists for the vaccine strains to be introduced into areas previously free of them [14,15,16,17]. Fourth, there are significant logistical challenges in distribution of a vaccine which requires storage in liquid nitrogen until administration into target animals. Fifth, the product has not been taken up by a private commercial organisation, which would be required to maintain sustainable distribution channels. Finally, there is the hope of a more conventional subunit vaccine, for which research is ongoing, that would be easier to manufacture and deliver (P. Spooner, personal communication). The institutional history that has formed the backdrop to this fascinating story was recently documented by Perry [3].

In order to be able to recommend the use of a vaccine in animal disease control strategies, knowledge of the detailed characteristics and capability of the vaccine are required, in addition to in-depth knowledge of the disease and its epidemiology [18]. Typically, official vaccine registration involves thorough objective assessment, which includes the demonstration of both the safety and efficacy, with relevant regulatory approval granted [18]. The use of field studies can allow the assessment of safety and efficacy under “real-life” conditions and can identify reactions (including mortality) not otherwise observed in a laboratory setting. However, the real-life nature of field studies presents an array of uncontrollable variables, creating a challenge in attaining high-quality controlled efficacy data, whereas the demonstration of safety is considered more reliable [18].

The Muguga cocktail was registered for use in cattle in Kenya, Tanzania, and Malawi between 2007 and 2009. The construction of the base registration dossier and the registration process were recently described by Peters et al. [19]. However, since no purpose-designed registration studies had been carried out, the evidence for safety and efficacy used in the dossier was based solely on a review of the readily available clinical literature. It has been widely accepted that although the Muguga cocktail ITM procedure has a narrow therapeutic index, animals receiving the vaccine apparently acquire life-long immunity to subsequent disease (see review by Morrison and McKeever [9]). However, despite many studies both in the laboratory and in the field, there has been no exhaustive systematic review of the Muguga cocktail ITM clinical safety and efficacy.

The systematic review process aims to identify all relevant studies in order to address a specific research question [20,21]. In systematic review of animal studies, meta-analysis is intended to assess the overall size and direction of an intervention’s effect, as well as examining possible sources of heterogeneity [22]. As it is an effective means of increasing power, meta-analysis can be particularly beneficial in animal studies which are often small and can, therefore, be underpowered [23]. Pooled estimates of the overall effect of an intervention can result in alternative conclusions of the safety and or efficacy of an intervention compared to qualitative assessment of individual studies [24,25].

This review seeks to establish the efficacy and safety of the Muguga cocktail vaccine by examining the literature and collating and analysing all relevant studies. It is hoped that the review will then assist researchers, policymakers, and livestock keepers to make informed decisions with regard to vaccine use in ECF control programs.

## 2. Materials and Methods

### 2.1. Protocol

Planned methods were documented a priori in a protocol detailing the proposed approaches for the review. There is not yet a standard protocol format for the registration of animal study systematic reviews [26]; therefore, a protocol format developed by de Vries et al. [26], based on the Cochrane review protocol [27] and the preferred reporting items for systematic reviews and meta-analyses (PRISMA) checklist [28], was used for this review. This standardised protocol format is supported by Collaborative Approach to Meta Analysis and Review of Animal Data from Experimental Studies (CAMRADES) and the SYstematic Review Centre for Laboratory animal Experimentation (SYRCLE), the largest supporting groups involved in systematic review of data from animal studies. The protocol was completed at the beginning of the review process and was approved by both authors. It was not possible to prospectively register/publish the protocol; however, it is included in Supplementary File S1. The PRISMA checklist is also included, in Supplementary File S2. This review did not require ethical approval.

### 2.2. Review Research Questions

The primary research question that formed the basis for the systematic review was

- What is the safety and efficacy of the Muguga cocktail vaccine?

A secondary research question was formulated following the population, intervention, comparison, and outcome (PICO) scheme. Due to the live nature of the vaccine, there is the requirement for concurrent treatment with long-acting oxytetracycline (OTC LA) to reduce the severity of the vaccine reaction, and as such, the secondary question was

- What is the safety and efficacy of oxytetracycline?

The key elements of the research questions are detailed in reference to the PICO scheme (Table 1).

### 2.3. Eligibility Criteria 

Studies from all dates were included. The search included peer-reviewed journal articles, data from published research reports and unpublished research reports (from expert sources), theses, conference proceedings, and field study reports. Challenge trials, field trials, and observational studies were included.

Due to time constraints, only articles written in English were included. If abstracts or full-text articles were not available, they were excluded. Where there was more than one report of an individual study’s results, the preferred version only was included so as to avoid duplication of results within analyses. Where there was discrepancy or ambiguity in data and or interpretation of results, attempts were made to contact authors to seek clarification, and discussion between the authors was initiated until agreement was reached, including the decision to include or exclude unclear studies.

### 2.4. Scoping Review

A preliminary search was carried out in March–April 2021 to trial search terms and strings, and to gauge the volume of available evidence. Search terms were reformatted and reassessed until a consensus was achieved.

### 2.5. Database Sources

A variety of electronic databases were used, including Web of Science, PubMed (Medline), CAB Direct, and Google Scholar; these databases being considered to cover the majority of veterinary literature [29]. As the geographic context of the systematic review is in Eastern, Central, and Southern Africa, the African Journals Online database was also searched, as it is a source of abstracts from more than 200 African journals and has links to more than 80 full text articles [30]. 

### 2.6. Search Strategy

Literature searches were conducted for all available years in Web of Science, PubMed (Medline), CAB Direct, Google Scholar, and African Journals Online in April 2021. References of relevant articles were searched (forwards and backwards citation tracking) to identify articles that may have been missed.

Search terms were standardised across databases to ensure comparable searches. Search terms were “East Coast fever OR ECF AND Muguga Cocktail OR Muguga AND vaccine”. In the case of “ECF ITM vaccine OR Muguga cocktail”, this string yielded the most studies, with the exception of African Journals Online (Table 2). Terms for outcomes were not included in the search (e.g., efficacy, safety, mortality, reaction, reactor) as it was thought that if they were not used clearly in eligible studies, it could result in studies being missed in database searches [31]. 

### 2.7. Study Selection

For all search terms, study titles were reviewed/screened for relevance to the Muguga cocktail vaccine specifically. Relevant title abstracts were then reviewed (by one author, F.A.) according to the selection criteria (Table 3), and articles were selected for full-text review. Full-text articles were reviewed by F.A. and a sample reviewed by A.P. to ensure agreement on inclusion. Where the same data were presented in more than one study article, only the most detailed version was included.

### 2.8. Data Management

References were managed in referencing software Mendeley (version 1.19.8, Elsevier, London, UK). For meta-analysis, studies were exported from Mendeley as a ris. file and imported into review manager software RevMan5 [32].

### 2.9. Data Extraction

Data were extracted from all studies considered for inclusion in the systematic review. Selected qualitative and quantitative metadata were extracted into a piloted Excel spreadsheet format. Data extracted included author(s), citation, year of study, country of study, cattle breed, production system, agro-ecological zone (AEZ), study type, randomisation, sample size, vaccine dose, oxytetracycline dose, vaccine efficacy (efficacy is generally determined quantitatively by calculation as described in Thrusfield and qualitatively as “the extent to which a procedure produces beneficial results under ideal conditions” [33]), seroconversion, onset of immunity, duration of immunity, ECF diagnosis method(s), and vaccine safety (safety is defined as “lack of local or systemic reaction, and in statutory tests usually involves daily observations for a 14-day period post-vaccination” [19,34]) (Supplementary Files S3 and S4). 

### 2.10. Data Analysis

Studies were grouped based on the available evidence for safety and efficacy and reviewed under the major subheadings (parameters) as considered relevant (and for the most part, as detailed in a European dossier) (Table 4). As already mentioned, included in the safety and efficacy of an ITM vaccine is the safety and efficacy of oxytetracycline LA, the evidence for which was also synthesised in this review. 

Included studies were assessed for bias using the Risk of Bias tool (RoB) created by SYRCLE for animal intervention studies [35], based on the Cochrane Collaboration RoB tool [36] and adapted for specific aspects of bias involved in animal intervention studies. Biases addressed were selection, performance, attrition, detection, and reporting bias.

Where meta-analysis was possible, the overall effect (Z) was calculated, as well as heterogeneity (I^2^) using RevMan5 software [32]. A *p*-value of <0.05 was considered significant. Binary variables were analysed in this review; therefore, odds ratios (OR) were calculated, along with their 95% confidence interval (CI). The weight of individual studies was obtained by calculating the inverse of the variation of the intervention effect (the larger the sample size of the study, the greater the weight). The use of a random- or fixed-effects model was based on heterogeneity; a fixed-effect model (Chi^2^) was used where heterogeneity was low, and where there was high heterogeneity, a random-effects model (Tau^2^) was used. Forest plots were generated as combined graphical and tabular presentation of individual studies contributing to meta-analysis. Study outcome effect measure and outcome uncertainty (confidence intervals), as well as the weight, are presented on the plot. 

Sensitivity analysis was conducted to determine robustness of observed meta-analysis outcomes. This involved repeating primary analysis with an altered dataset to assess whether there was an effect on the overall outcome effect.

## 3. Results

**Figure 1:**
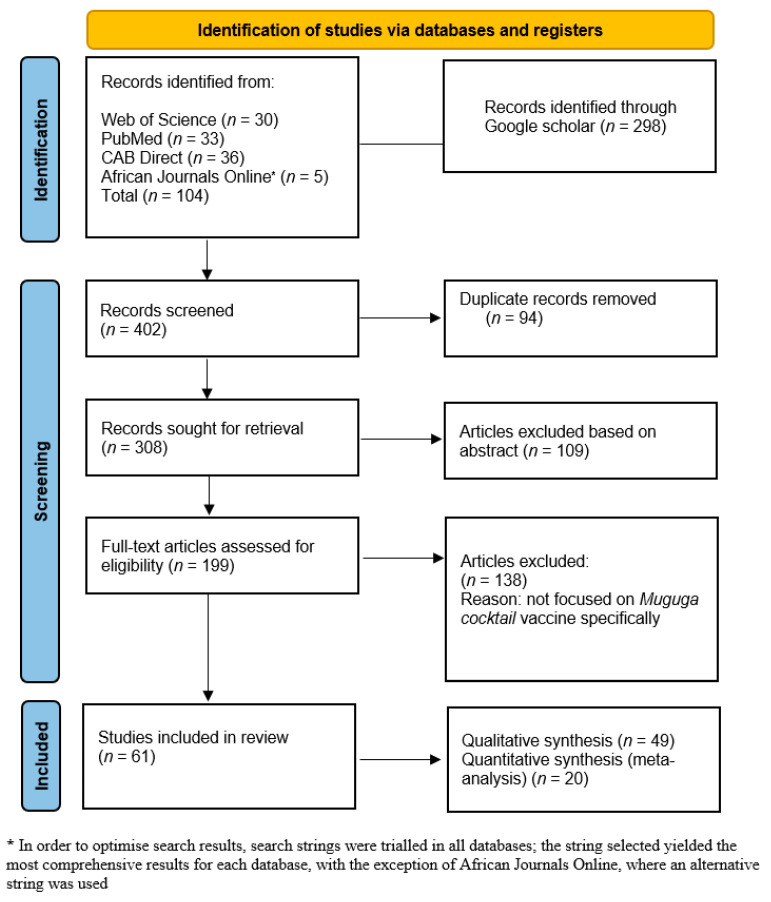
PRISMA flow diagram for article screening and inclusion (adapted from [28]). All 402 articles identified in the literature search were assessed as relevant. Ninety-four duplicates were removed, as well as a further 109 articles, based on abstract content. On full-text assessment, 138 articles were excluded, resulting in 61 articles to be included in the review.

The number of returns for each search is included in Table 2, providing an overview of the popularity of each term/string. Results of the literature search are presented in Figure 1. A total of 402 studies were identified through the database searches and reference lists. Duplicates were removed (*n* = 94), and 109 studies were excluded based on their abstracts. Of the 199 full-text studies assessed for eligibility, 61 were included in the review. 

Of the 61 selected studies for review, 54 specified the study country. Most studies were conducted in Tanzania (*n* = 22), followed by Kenya (*n* = 14), Uganda (*n* = 6), Malawi (*n* = 6), Zambia (*n* = 3), and Rwanda (*n* = 1). Single studies were conducted in Comoros, Democratic Republic of Congo (DRC), and Burundi.

Of the 61, 26 studies did not specify the year in which the work was carried out. Of the 35 that did specify year, the studies ranged from 1974 up to 2018.

### 3.1. Efficacy

#### 3.1.1. Protection against ECF Mortality in Immunisation Trials

Of the 61 studies included for analysis in the systematic review, 18 described mortality rates from immunisation field trials using the Muguga cocktail vaccine. Several of these studies presented results for more than one trial [12,37,38,39,40]; in these instances, each trial was included in analysis individually (identified with suffix a, b, c, or 1, 2, 3 in the case of Radley 1975c [12] (it should be noted that the stabilates used in this study were the three components of the Muguga cocktail but were not yet referred to as such)), resulting in 27 studies (a total of 30 studies presented controlled quantitative trial data; however, three of these could not be included in meta-analysis due to inadequate or unclear data [40] (trials a and c) [41]) for meta-analysis. The Uilenberg et al. [37] study presented three trials, one of which was also reported by Schreuder et al. [42] and one by Uilenberg et al. [43]; only the one trial not otherwise presented was included in the analysis, thus avoiding duplication of analyses.

**Figure 2:**
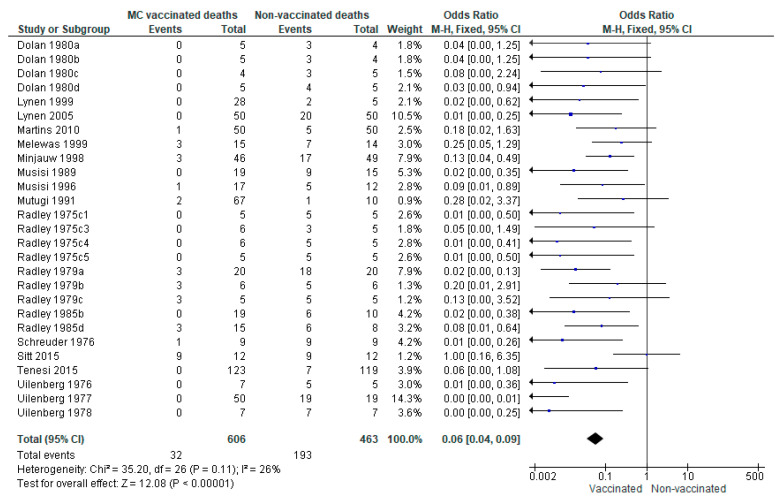
Forest plot showing comparison of mortality in Muguga cocktail (MC) vaccinated vs. nonvaccinated. The blue boxes represent the effect size (odds ratio) of each study—a bigger box indicates the study was weighted more, i.e., a larger sample size. The black diamond represents the pooled OR of the studies.

The outcome effect measure for vaccination on ECF mortality is expressed as odds ratio (OR); the pooled (black diamond) estimate of effect was observed to be 0.06 (95% CI: 0.04–0.09), and, as it did not cross the vertical line, shows a statistically significant effect favouring vaccination. The test for overall effect (Z) corroborates the results by presenting a *p*-value < 0.05. The studies were relatively homogeneous, with only 26% heterogeneity (I^2^) (Figure 2). As the studies were considered homogeneous (I^2^ < 50%), a fixed-effect model of meta-analysis was considered suitable.

Sensitivity analysis was conducted by repeating the primary analysis, excluding the study by Sitt et al. [44], based on the higher OR of 1 for outcome effect. In removing this study, the OR for overall effect decreased slightly to 0.05 (95% CI: 0.03–0.08), and the heterogeneity (I^2^) reduced to 6%.

##### Risk of Bias of Included Mortality Studies

For the eighteen studies that reported controlled quantitative data on ECF mortality, risk of bias was assessed using a tool developed by SYRCLE [35] (Supplementary File S5). It is not recommended that a summary score be calculated for studies so as not to assign weight to individual domains. Instead, a conservative approach was taken by the authors to provide an overall summary per study based on all domains. The majority of studies were assessed as high risk of bias, with one study considered moderate risk, and one considered low risk of bias.

#### 3.1.2. Efficacy in Response to Experimental Challenge

Specific challenge studies are a key component of the measure of efficacy and are a critical step in determining potency during manufacture of Muguga cocktail. Reactions in immunised and experimentally challenged animals are based on clinical and parasitological parameters combined to create a gradation, and have been classified by Anon [45] as no apparent reaction (NR) (no schizonts detected and no clinical signs observed); mild reaction (MR) (few schizonts detected, no fever or only low grade for a few days, and otherwise clinically normal); moderate reaction (MOR) (schizonts detected, persistent fever and transient clinical signs but recovers); severe reaction (SR) (high schizont parasitosis, persistent fever >9 days, clear clinical signs and may recover or die).

**Figure 3:**
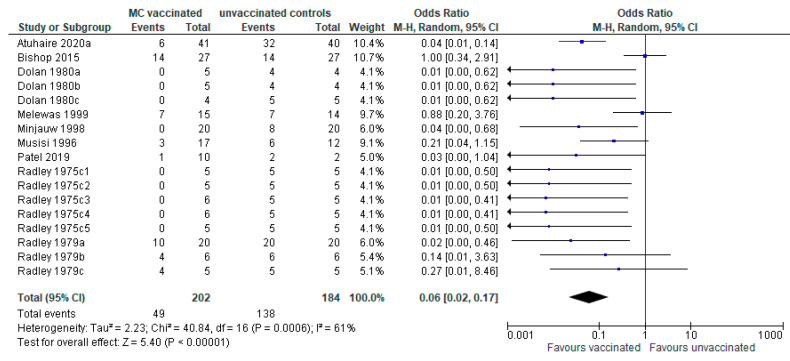
Forest plot showing comparison of severe reactions in MC vaccinated vs. nonvaccinated. The blue boxes represent the effect size (odds ratio) of each study—a bigger box indicates the study was weighted more, i.e., a larger sample size. The black diamond represents the pooled OR of the studies.

It was possible to conduct a meta-analysis of nine studies (seventeen trials) describing severe reactions to challenge in vaccinated and unvaccinated animals (Figure 3). As well as reporting reactions to challenge, Atuhaire et al. [46] also recorded severe reactions following vaccination, as a measure of vaccine viability. Severe reactions were compared to the ECF reaction index scores [47]. Similarly, Melewas et al. [48] also reported reactors to immunisation (3.9%; 576 of 14,628). They did not report severity but did report that four animals succumbed to anaphylaxis after immunisation (0.03%).

The outcome effect measure for vaccination on severe reactions is expressed as OR: the pooled estimate of effect was observed to be 0.06 (95% CI: 0.02–0.17), showing a statistically significant effect favouring vaccination. The test for overall effect (Z) corroborates the results by presenting a *p*-value < 0.05 (*p* < 0.00001). Heterogeneity (I^2^) for the studies was 61%, where <50% is considered homogenous, and so a random effect model of meta-analysis was suitable. 

Sensitivity analysis was conducted by repeating the primary analysis, excluding Bishop et al. [49] due to the higher OR of 1 for outcome effect. In removing this study, the OR for overall effect reduced slightly to 0.05 and the heterogeneity (I^2^) reduced to 37%. Similarly, reanalysis without Melewas et al. [48](OR = 0.88, CI: 0.20–3.76) also resulted in the overall effect OR reducing slightly to 0.05 and the heterogeneity reducing to 56%. 

##### Risk of Bias of Severe Reaction Studies

For the nine studies that reported controlled quantitative data on severe reactions to challenge, risk of bias was assessed (Supplementary File S6). Overall, the studies were all considered as high risk of bias.

#### 3.1.3. Efficacy Based on Seroconversion as a Measure of Protection

Although the presence of serum antibodies does not necessarily reflect protection against ECF, it does indicate exposure to the parasite. Some studies have accordingly used seroconversion as an indicator of vaccine response.

Ten studies presented seroconversion rates. Anon [50] observed very high rates of seroconversion by 30 days—93.9% in those given 1:80 vaccine dose, and 84% seroconversion with 1:100 dose. Anon [51] described 95% seroconversion with batch FAO-2 and 100% with FAO-1, by day 45 in both batches.

Atuhaire et al. [46] reported 35 of 41 immunised (85.4%) seroconverting, and Kiraithe [52] reported 69% seroconversion in vaccinated calves in Mutara and 83% in Ole Naishu (compared to 6.2% and 3.1% of controls, respectively). Kazungu et al. [53] reported significantly higher seropositivity in vaccinated cattle compared to unvaccinated. Six of the 381 (1.6%) cattle in the study were seronegative, one of which was vaccinated. A significant positive association between seropositivity and time since vaccination was reported.

Martins et al. [54] observed 60% seropositivity by 180 days in calves vaccinated between 1–2 months of age, and 80% seropositivity when vaccinated over two months of age, i.e., suggestive of relatively good rates of seroconversion. Mbassa et al. [55] compared storing the vaccine at −70 °C for six weeks and six months. They showed that even when stored at −70 °C for six months, all immunised animals that were tested (*n* = 38) had seroconverted by day 30, i.e., 100% seroconversion in the tested group.

Patel et al. [56] observed a range of seroconversion rates in 62 immunised animals, from 75% to 87%, with a mean of 82%. Turasha [57] also reported a range of seroconversion rates, from 55.6% to 79.4%, with a mean of 69.2%. The report described mean prevaccination seroprevalence of 19.9% compared to 69.6% post-vaccination, remarking on good overall seroconversion [57]. 

Tenesi [58] observed 93% seroconversion post-vaccination (increased significantly from 46% prevaccination), compared to 42% seroconversion in control animals. Wesonga et al. [59] presented seroconversion rates for several study sites in Kenya; in Mutara, prevaccination seropositivity was 3%, compared to 69% in post-vaccination, and for Ole Naishu, there was 1.6% seropositivity prevaccination compared to 83% post-vaccination. In control animals for Mutara, 4.7% were seropositive at day zero compared to 6.3% at day 35, and in Ole Naishu, there were 3.1% seropositive controls at both day zero and at day 35. The study, therefore, demonstrated a high proportion of seroconversion after vaccination in Ole Naishu (80–90% considered acceptable) and lower than acceptable seroconversion in Mutara.

#### 3.1.4. Efficacy as a Percentage (Quantitative)

Seven studies described Muguga cocktail vaccine efficacy as a percentage. Four studies described the fraction of severe reactors in control and vaccinated groups to calculate vaccine efficacy, as described by Thrusfield [33]. One study calculated efficacy as proportion of positive animals to total number of animals, and two studies did not state how efficacy was calculated.

Kiraithe [52] observed 97.8% and 78.4% efficacy in two separate ranches in Laikipia County, Kenya. Vaccine efficacy was calculated using incidence rate, as described in Dohoo et al. [60]. Similarly, Lynen et al. [61] described 97.6% efficacy in preventing ECF cases and 97.9% efficacy in preventing ECF deaths. Martins et al. [54] reported 97% efficacy in Tanzanian pastoralist systems, with significantly fewer cases of ECF in the vaccinated group compared to the unvaccinated. More recently, Tenesi [58] reported vaccine efficacy of 89.4% in Narok County, Kenya.

Magwisha et al. [62] reported overall efficacy of 70%, based on the proportion of positive animals (*n* = 853) to total animals (*n* = 1216), whereby antibody titre post-immunisation ≥20 PP was considered the threshold for protection. This is a somewhat tenuous link to efficacy; as already stated, seroconversion does not equate to efficacy per se, but rather indicates a vaccine response.

Following success in Malawi, Tanzania, and Zambia, the Muguga cocktail vaccine was trialled in Uganda between 1990 and 1993, with 80% protection against local parasite challenge [63]. Another report of Muguga cocktail immunisation in Uganda, around the same time, reported 2005 animals vaccinated and 86% protection [64].

#### 3.1.5. Onset of Immunity

As for previous sections, seroconversion does not directly equate to immunity, however, it is a widely accepted indirect indicator of a protective response. Six studies reported onset of immunity with regards to vaccine efficacy. Anon [50] observed a high proportion of seroconversion by 30 days (93.9% for those given 1:80 vaccine dose, and 84% in those given 1:100 dose) and no reactions with either vaccine dose. Anon [51] similarly described a high rate of seroconversion, by 45 days (95% vaccinated with batch FAO-2 and 100% vaccinated with batch FAO-1). Anon [65] reported seroconversion between 28 and 35 days. Kiraithe [52] observed seroconversion by day 35 in the majority or vaccinated calves (69% in Mutara and 83% in Ole Naishu), and only very few controls (6.2% and 3.1%, respectively).

Oura et al. [66] described seroconversion by day 48. Sitt et al. [44] reported seroconversion in all but one vaccinated calves, between days 21 and 35, whereas the controls remained negative by enzyme-linked immunosorbent assay (ELISA).

#### 3.1.6. Duration of Immunity

A single study reported on duration of immunity. Oura et al. [66] described the presence of antibodies to *T. parva* by ELISA at day 48 post-vaccination.

#### 3.1.7. Efficacy as a Statement (Qualitative) 

Six studies described vaccine efficacy in qualitative terms. Akoolo et al. [67] reported solid resistance to challenge with a potentially lethal dose of vaccine batch FAO-1 after vaccination with FAO-1. Anon [68] described a “very high degree of vaccine efficacy”, based on a 90% reduction in ECF morbidity/mortality in ECF endemic areas following immunisation with vaccine batch FAO-1 in Tanzania.

Homewood et al. [69] reported a “highly significant impact on survival” following use of the ITM vaccine (this study did not specify that the ITM vaccine in use was Muguga cocktail, however communication with the authors via email confirmed Muguga cocktail was indeed used). The study found the probability of a vaccinated animal surviving to 54 months was 97%, in comparison to 71% for those unvaccinated, and this reduced calf mortality due to ECF from more than 20% to around 2%.

A titration study by Mutugi et al. [70] described direct evidence of efficacy of the optimal 1:80 dose of FAO-1 stabilate against experimental challenge, stating “a good margin of vaccine efficacy exists as shown by the unequivocal good result of the 1:100 dilution”. Patel et al. [71] conducted a dose determination study and concluded that 1 ml of a 1:100 vaccine dose (ILRI-0804) was considered efficacious (as well as safe—discussed later).

Mutugi reviewed the safety and efficacy of the Muguga cocktail and reported that “the efficacy of the FAO-1 batch is exemplified by the successful immunisation of some 400,000 cattle in Uganda and Tanzania in recent years with a high and rising demand for the ITM technology” [72]. A high degree of efficacy was reported with more than a 90% reduction in ECF morbidity and mortality following immunisation with the FAO-1 vaccine in Tanzania between 1998 and 2007.

The third of the early Radley et al. studies [12] compared immunisation with a combination of theilerial strains (Muguga, Kiambu 5, and Serengeti transformed) to immunisation with only one or two strains separately, followed by heterologous challenge. Inapparent or mild reactions were observed in those animals immunised with the combination (Muguga cocktail components), whereas both the controls and those immunised with only one or two strains had severe reactions or died. Additionally, when cattle immunised with the combination of strains were challenged with a lethal dose of three heterologous *T. parva* strains, inapparent or mild reactions were observed, compared to the control animals, almost all of which (13/15) died.

Steinaa et al. [73] compared the protection given with a single *T. parva* strain (Muguga) to immunizing with the trivalent Muguga cocktail, and found no substantive difference in protection, with no broader a CTL response when using the cocktail, suggesting limited antigenic diversity in the cocktail.

#### 3.1.8. Efficacy near Buffalo

Ten studies reported the apparent efficacy of the Muguga cocktail vaccination in cattle in proximity to buffalo; four studies reported the vaccine to be efficacious at this interface, two studies observed efficacy to be reduced or partial, and four studies (three of these studies report on the same dataset [49,74,75]) reported inefficacy. 

Di Giulio et al. [13] reported on the several studies of vaccine uptake and environmental impact in northern Tanzania, including longitudinal studies in pastoral communities, where vaccine efficacy had been observed in areas including livestock–buffalo interfaces; 92% reduction in overall calf mortality was seen in the Ngorongoro Conservation Area (NCA) where there is a high degree of livestock–wildlife interaction, and 95% reduced calf mortality was observed in the Tanzania-Kenya border study areas (considered to be unstable ECF-endemic). Lynen et al. [76] also reported clear demonstration of Muguga cocktail vaccine efficacy in pastoral farming systems in northern Tanzania, including buffalo areas. There were no deaths reported due to ECF in vaccinated cattle (n = 50) compared to 25 deaths due to ECF in those nonvaccinated (50%).

As already mentioned (efficacy as a percentage), Martins et al. [54] conducted a study to assess the impact of immunisation with the Muguga cocktail, observing 97% vaccinal efficacy in Endulen, NCA; although the study does not make reference to buffalo, their presence in this area is highly likely. In a report by Turasha [77], it was concluded that the Muguga cocktail vaccine was both safe and protective of cattle in pastoral systems in Kenya, and was protective even where buffalo were present (Loita division).

Kiraithe [52] reported vaccine efficacy of 97.8% in Ole Naishu, Kenya, where contact with buffalo was minimal, in comparison to Mutara, Kenya, where buffalo and cattle shared grazing and the vaccine efficacy was lower, at 78.4%. Several possible explanations for the varied efficacy were given, including level of tick challenge, tick infection rates, environmental conditions, and interaction with wildlife, in particular buffalo. 

As included in the efficacy meta-analysis (protection against ECF mortality), Radley et al. [38] conducted challenge trials whereby Muguga cocktail-immunised cattle were exposed to *T. lawrencei* tick challenge in a buffalo paddock (experiment 2). All six control animals underwent severe reactions and five died of theileriosis, whereas four of the six immunised animals underwent severe reactions, with three succumbing to theileriosis. Similarly, in experiment 3, four Muguga cocktail-immunised cattle exposed in the buffalo paddock underwent severe reactions, with three of them fatal, compared to all five controls succumbing to fatal *T. lawrencei* infections; thus, although demonstrating some protection, field protection was inadequate. 

A series of studies refer to a vaccine field trial conducted in Kenya in 2000, whereby 113 animals were challenged with *T. parva* from buffalo-associated ticks; 40 immunised cattle and 13 control cattle developed clinical disease (mostly typical of buffalo-derived *T. parva*) and died [49,74,75]. The Muguga cocktail FAO-1 was one of three vaccine stabilates trialled (also Marikebuni stabilates 316 and 3014); in the 27 animals immunised with the FAO-1, there were 14 severe reactions, which was the same outcome in the control group (14/27 severe reactions). Pelle et al. [74] conducted genotypic analyses using samples from the vaccines trial, and although they did not present clinical reaction data from the trial, their findings were consistent with immunisation studies demonstrating that immunity induced by a mix of parasites is not always effective against buffalo-derived challenge.

Sitt et al. [44] observed that both vaccinated and unvaccinated cattle had the same number (9/12) succumb to *T. parva* when grazed in a site with buffalo only (no other cattle) at Ol Pejeta, Kenya, and thus the vaccine did not affect the survival outcome. Additionally, there was no significant difference in onset or progress of disease in vaccinated and unvaccinated cattle. The study demonstrated that the Muguga cocktail did not protect against buffalo-derived *T. parva.*

#### 3.1.9. Efficacy and Storage Temperature

Two studies assessed how temperature affected vaccine efficacy. Standard medium for storing and transporting *T. parva* stabilate for ITM vaccination is liquid nitrogen (−196 °C) [19]. Atuhaire et al. [78] recently investigated vaccine efficacy when Muguga cocktail stabilate was transferred from liquid nitrogen to −80 °C for up to 30 days, and reported no significant effect in its ability to infect cattle (or cultured PBMCs), thereby suggesting that dry ice (−79 °C) could be a suitable alternative cold chain. Mbassa et al. [55] also observed that transferring stabilate from liquid nitrogen to −70 °C maintained vaccine efficacy over a period of six months, with only a slight time-dependent reduction in vaccine potency.

### 3.2. Safety

#### 3.2.1. Safety as a Statement (Qualitative)

Ten studies reported on vaccine safety in qualitative terms (Table 5). 

#### 3.2.2. Shed and Transmission

Thirteen studies reported the presence of Muguga cocktail vaccine components in unvaccinated cattle (or nontarget animals), with the possibility of sharing of stocks via tick transmission and unrestricted movement/nomadism of cattle in the region. All studies reporting possible shed and spread are shown in Table 6.

### 3.3. OTC Safety and Efficacy

A potential confounding factor in the safety and efficacy of the ITM vaccine is the long-acting oxytetracycline (OTC) used to control the clinical response to infection, as there is a variety of OTC formulations. Radley et al. [10,11,12] first described the use of oxytetracycline (OTC), with a series of daily injections, before the availability of a long-acting (LA) formulation. A series of studies used a 20 mg/kg OTC LA dose rate in determining the optimal therapeutic vaccine dose; however, there were reports of some adverse clinical reactions to vaccination [19]. The OTC LA dose rate was increased to 30 mg/kg, but the volume was sometimes very high using the 20% formulation; a 30% formulation became available, and both the frequency and severity of vaccination reactions were reduced when using 30 mg/kg. Lynen et al. [92] conducted a series of experiments using two OTC LA formulations (20% and 30%) and observed significant reductions in both mild and severe reactions when using the 30% formulation (7.1% mild reactors with 30% compared to 21.4% with 20%, and 7.4% severe reactors with 30% compared to 44% with 20%). Additionally, vaccine reactors reduced in the field, from around 15% to less than 1%, after the 30% formulation was introduced. Anon [50], Anon [51], and Anon [68] also reported preferential use of the 30 mg/kg formulation.

A study by Clarke et al. [93] was conducted to compare the pharmacokinetics of two long-acting OTC preparations, manufactured by Boehringer Ingelheim and Merial, respectively. Both preparations were given at the same dose (20 mg/kg) via the subcutaneous or intramuscular route in Hereford steers. The pharmacokinetics in all four settings (two manufacturing brands, two routes of administration) were very similar, with only marginal differences, concluding that both OTC products were bioequivalent.

A study to compare 30% OTC LA given at 30 mg/kg and 40 mg/kg observed that there were fewer reactions with the higher dose, but there were also fewer vaccine seroconversions, and as such, the 30 mg/kg dose was considered optimal [94].

## 4. Discussion

Veterinary vaccines have brought significant benefits to animal health and production, with research advances bringing about safe and more effective vaccines [95]. This review aimed to examine the evidence regarding the safety and efficacy of the Muguga cocktail vaccine, an unconventional vaccination procedure to protect cattle from ECF. A total of 61 studies met the criteria to be included in the systematic review. It was possible to conduct meta-analyses for ECF mortality in vaccinated and control animals, as well as for severe reactors in vaccinated and control animals. Other studies were synthesised in narrative summaries.

Results of the meta-analysis of ECF mortality showed a significant effect favouring vaccination (OR: 0.06; 95% CI: 0.04–0.09, *p* < 0.00001). With all 18 studies (27 trials) included in the meta-analysis, heterogeneity was relatively low at 26%; however, following sensitivity analysis by excluding the Sitt et al. [44] study (OR = 1), heterogeneity reduced greatly to only 6%, indicating a highly homogenous dataset, i.e., the studies were similar. Meta-analysis of severe reactors to challenge also showed a significant effect favouring vaccination (OR: 0.06; 95% CI: 0.02–0.17; *p* < 0.00001). There was heterogeneity observed in the overall effect (61%); however, this was reduced to 37% when the dataset was reanalysed with Bishop et al. [49] excluded, and reduced to 56% when Melewas et al. [48] was excluded, suggesting differences in these studies from the others. Possible differences include the buffalo-associated tick challenge in the Bishop et al. [49] study potentially resulting in the vaccine being less efficacious, and in the Melewas et al. [48] study, the trial was conducted in August–September, considered the season of high ECF challenge. 

Evidence of vaccine efficacy was synthesised, both quantitatively and qualitatively. For the seven studies presenting calculated percentage efficacy, not all studies calculated efficacy correctly (as defined by Thrusfield [33]). The percentage efficacy reported, however, ranged from 70 to 97.9%. Efficacy was also reported qualitatively by eight studies. The majority (seven of eight) of studies reported the Muguga cocktail to be efficacious, with statements such as “very high degree of vaccine efficacy” [68], “good margin of vaccine efficacy” [70], and “efficacy is exemplified by the successful immunisation of some 400,000 cattle in Uganda and Tanzania in recent years” [72]. A single study by Steinaa et al. [73] reported no substantive difference in protection with the Muguga cocktail in comparison to a single *T. parva* strain (Muguga), suggesting limited antigenic diversity in the cocktail. Although not stated explicitly, efficacy was implied. 

***Efficacy based on seroconversion.*** Seroconversion following immunisation is indicative of vaccine viability [55] but not necessarily efficacy. A viable vaccine administered properly is expected to induce seroconversion of 80–90% [59]. The ten studies that described seroconversion rates reported figures from 55.6% to 100% seroconversion; thus, not all studies reported seroconversion within the range considered acceptable for a viable vaccine. Wesonga et al. [59] reported that the inadequate seroconversion observed at one of the study sites (Mutara) was likely due to the viability of the vaccine having been reduced during storage or transfer and that, despite careful handling by experienced vaccinators, it is thought that this is a common occurrence in the field. Poor handling has been shown to influence the efficacy of the vaccine, and seroconversion <70% is thought to be indicative of poor handling, e.g., poor inoculation technique or cold chain issues [59]. 

***Proximity to buffalo*.** Studies were analysed with regards to vaccine efficacy specifically in proximity to buffalo. It has been shown in cattle co-grazed with buffalo that there is greater antigenic diversity in buffalo-derived *T. parva* and that the cattle-maintained parasite population is a subset [74,87,96,97,98,99], which is thought to affect the efficacy of the vaccine (with limited diversity) at the cattle–buffalo interface. Ten studies reported use of the Muguga cocktail in areas where buffalo were known to be present. Four of the studies reported the Muguga cocktail to be efficacious even when used in areas with buffalo [13,54,76,77]. Two studies reported reduced or partial protection [38,52], and four studies observed ineffective protection when the vaccine was used to protect against buffalo-derived challenge, including the three studies sharing the same samples from a cattle–buffalo interface [44,49,74,75]. Reduced efficacy in proximity to buffalo was also demonstrated inadvertently in the mortality meta-analysis, whereby the outcome effect of the Sitt et al. [44] study had a higher OR compared to other studies, and its removal from the meta-analysis reduced the heterogeneity greatly. There are no readily apparent explanations for the varied vaccine efficacy observed in the studies, but it is likely that the environmental conditions, degree of tick challenge and tick infection rates, parasite population genetics, and level of interaction with wildlife all play important and interconnected roles.

***Immunity onset and duration.*** Evidence of onset and duration of immunity were assessed as parameters of vaccine efficacy. In the six studies reporting onset of immunity, seroconversion was observed between day 28 and day 48, with most studies reporting seroconversion by day 35. With regard to duration of immunity, it is generally considered that lifelong immunity is induced in vaccinated cattle [9]; however, only a single study reported duration of immunity.

The final parameter considered was vaccine storage temperature and vaccine efficacy. Atuhaire et al. [78] observed no significant effect when Muguga cocktail stabilate was stored at −80 °C for 30 days. Similarly, Mbassa et al. [55] observed −70 °C maintained vaccine efficacy over six months. These studies indicate that dry ice could be used in an alternative cold chain.

***Vaccine safety.*** Equally important to vaccine efficacy is vaccine safety. A reactor rate of 0.5% (5 in 1000) is regarded as acceptable [57]. Ten studies reported vaccine safety qualitatively, with nine of them reporting the Muguga cocktail to be considered safe, with statements such as “very safe for field conditions”, “concluded even doses of 1:10 were safe”, and “safe in terms of survival and few reactions”. A single study by Mbyuzi et al. [80] reported mortality in newborn calves from vaccinated dams, stating that further safety evaluation was required. Due to the lethal nature of the vaccine stocks, the severity of reactions tends to reduce the safety margin, which is thought to reduce its uptake [55] as farmer confidence of the vaccine is affected [57]. 

Thirteen studies described the persistence of vaccine components in unvaccinated (nontarget) animals. The possibility of shedding into the environment, and cross infection of nontarget animals, can lead to unintended severe adverse events (SAEs) [100], and transmission of vaccine components into the field has understandably caused concern that recombinant parasite populations could be established against which the Muguga cocktail is not effective; however, it is also possible that vaccine antigens in field parasites could augment vaccine effectiveness [13]. Whilst there may indeed be a risk of transmission, it is largely speculative and is not specific to vaccination, with the potential to occur in naturally infected animals as well, and after four decades of use, there has been no such occurrence of SAEs reported. 

***Safety and efficacy of OTC.*** As concurrent administration of OTC is essential with the ITM vaccine, safety and efficacy of OTC were also assessed in this review. It was reported that 30 mg/kg OTC was optimal [94] and 30 mg/kg was reported as preferred and more effective [92]. Three further studies also reported preferential use of 30 mg/kg [50,51,68]. 

***Risk of bias.*** Bias is an essential consideration in conducting systematic reviews. Almost all of the included studies were judged overall as high risk of bias, based on selection and performance biases, although generally studies had low risk of bias for attrition and reporting. As well as from the individual studies, there is always the risk of bias in the results of meta-analyses if there is the absence of studies that should have been included [101]. Systematic review can fail to identify all eligible studies, for example, those in inaccessible publications, resulting in publication bias, or studies may present the most significant results, resulting in reporting bias [102]. The low risk of reporting bias is suggestive that there is not an absence of studies or results that should have been included in this review.

Many signalling questions were judged as “unclear” risk of bias. This was not expected; however, reporting of experimental methodology details in animal studies is considered poor [103]. This highlights the requirement for improved methodology in animal studies, or at least improved reporting of methodological details. For example, animal weight is an essential requirement in order to administer the correct dose of ITM vaccine, and therefore weights must have been recorded, yet very few studies reported this baseline characteristic. In general, the more recent studies tended to report study methodology more thoroughly than the older studies.

***Study limitations.*** There are numerous difficulties in animal challenge studies, including ensuring the “three Rs rule”, to replace, reduce, and refine animal tests wherever possible [18] and, as such, sample sizes are often very small. Statistically significant data from field efficacy studies can be difficult to obtain, and due to uncontrollable outside variables, these studies can be inconclusive. As such, it can be necessary to combine laboratory and field study efficacy data in order to demonstrate product efficacy, with a posteriori field studies linked to vaccine surveillance [18] or vaccinovigilance [104]. There were numerous uncontrolled variables in study design, and study design features were often not reported, including time of year (with respect to seasonality of ECF), vaccine batch and dose, and OTC dose. Challenge stabilates were also highly varied, often using local stocks. While these study design features could not be controlled for, they do, however, represent a real-life situation, where animals are challenged with local *T. parva* populations and at varying times of the year. 

Several factors influence vaccine efficacy, including host, human, environmental, and intrinsic vaccine factors [95], and factors of vaccine safety go beyond manufacture, testing, and trials, and include safe vaccine transportation, administration, and surveillance [105], as demonstrated in this review. The positive impact of the vaccine on livelihoods has been demonstrated in studies such as Homewood et al. [69], where calf mortality was reduced significantly. However, it was also observed that poorer livestock keepers vaccinated only a small proportion of their younger stock compared to wealthier farmers. It is thought that this variation in livelihood impact was due to cost as well as vaccine pack-size discriminating against the smallholders. While the economic benefit of the Muguga cocktail vaccine was not included in this review, it has been said that it offers a less expensive and more effective control option, without dependence on costly acaricides [106]. 

## 5. Conclusions

The studies in this review present the available evidence for safety and efficacy of the Muguga cocktail vaccine. Overall, the evidence—both quantitative and qualitative—indicates that the vaccine is both safe and efficacious, although the review did identify some areas of uncertainty. With regards to safety, the study by Mbyuzi et al. [80] was the only apparent study with data on use of the Muguga cocktail in pregnant animals. It is anticipated that many pregnant (and lactating) animals must have been vaccinated with no safety concerns reported; however, this is an area requiring further research. The presence and persistence of vaccine components in nonvaccinated animals require further genotyping studies and, importantly, molecular epidemiological surveillance studies. With regard to efficacy, the reduced vaccine efficacy sometimes reported in cattle in proximity to buffalo also requires further comparative analyses of the population genetics of *T. parva* populations in sympatric cattle and buffalo in order to better understand the barriers to effective protection.

There is an inextricable link between livestock health and the health, wellbeing, and livelihoods of livestock keepers. Considering the magnitude of economic damage caused by theileriosis, vaccination with the Muguga cocktail ITM vaccine should be an accessible and affordable control option for even the smallest of livestock keepers, allowing for positive impact on animal health and production, and, importantly, livelihoods of all scales.

## Figures and Tables

**Table 1:** Key elements of the research questions as detailed by the PICO scheme.

PICO Scheme	Question Elements
Population	Cattle
Intervention	Immunisation with the Muguga cocktail ITM vaccine
Comparison	Nonimmunised (control) cattle
Outcome	Efficacy of Muguga cocktail vaccination (demonstrated as reduction in mortality and/or reduction in severe reactors in immunised compared to nonimmunised controls) and safety of Muguga cocktail and OTC (demonstrated by lack or local or systemic reactions in animals or the environment (including nontarget animals))

**Table 2:** Database search terms and results.

Search Term	Database	Number of Results
ECF ITM vaccine	Web of Science	12
	PubMed	12
	CAB Direct	13
	African Journals Online	1
Muguga Cocktail	Web of Science	24
	PubMed	26
	CAB Direct	27
	African Journals Online	4
ECF Muguga vaccine	Web of Science	14
	PubMed	16
	CAB Direct	26
	African Journals Online	5
ECF Muguga Cocktail	Web of Science	11
	PubMed	13
	CAB Direct	12
	African Journals Online	3
ECF ITM vaccine OR Muguga cocktail	Web of Science	30
	PubMed	33
	CAB Direct	36
	African Journals Online	0
	Google Scholar	296

**Table 3:** Study selection criteria for ECF Muguga cocktail vaccine.

Domain	Criteria
Date range	All
Geographical scope	Global
Type	Peer-reviewed journal articles, research reports including published and unpublished field studies, theses, conference proceedings. Challenge trials, natural infections, and observational studies.
Specific details	Reported efficacy and safety of use of ECF ITM (Muguga cocktail) vaccine
Exclusions	Abstract or full text unavailable
Language	English only

**Table 4:** Grouping of studies based on parameters relating to safety and efficacy issues.

Issue	Parameter
Efficacy	Efficacy (quantitative) demonstrated by mortality rates
	Efficacy in response to challenge (quantitative)
	Efficacy based on Seroconversion as a measure of protection
	Efficacy as a percentage
	Onset of immunity
	Duration of immunity
	Efficacy (qualitative)
	Efficacy in proximity to buffalo
	Efficacy and storage temperature
Safety	Safety (qualitative)
	Shed and transmission
Safety and efficacy of OTC	Safety and efficacy of OTC

**Table 5:** Studies describing safety qualitatively.

Study	Description of Safety
Anon 2007 [68]	Reported that vaccine FAO-1 was accepted as safe in Tanzania.
Anon 1998 [50]	The study compared 1:80 and 1:100 dilutions of FAO-1 with OTC LA 30 mg/kg and showed that both dilutions were very safe for field conditions, with no reactors encountered.
Anon 1999 [51]	Negligible reactors to either vaccine batch FAO-1 or FAO-2 with a 1:80 dilution and OTC LA 30mg/kg and were both confirmed as being safe.
ILRI 1996 [79]	Four groups of two cattle were immunised with FAO-1 vaccine dilutions of 1:10, 1:20, 1:40, and 1:80, with concurrent 20 mg/kg OTC (Terramycin LA 20%), followed by homologous challenge of all immunised as well as two control animals. None of the vaccinated animals underwent severe reactions, whereas the controls both reacted severely. It was concluded that even doses of 1:10 were safe.
Mbyuzi et al., 2013 [80]	Observed mortality of calves born from immunised dams and reported the requirement for further safety evaluation and verification.
Mutugi et al., 1997 [70]	Reported a 1:80 dose of FAO-1 to be safe, in terms of survival and few reactions.
Mutugi [72]	A review of the safety of the FAO-1 vaccine stated that “the refinement of the vaccine used has given greater confidence that the ITM procedure is safe and does not compromise productivity”. The review reported that the majority of the 37,000 cattle immunised in Uganda (1998–2007) showed inapparent or only mild reactions, with 2.7% (1000)undergoing severe reactions and five deaths (0.01%) associated. The 1:80 FAO-1 dilution used in Kenya “provided a bigger safety margin while at the same time being efficacious (than the 1:60 or 1:100)”, with a “very high degree of safety margin observed in the field”.
Patel et al., 2016 [71]	A safety and dose determination study was conducted using a three-stage immunisation and challenge trial (immunizing with ILRI-0804 Muguga cocktail and challenging with FAO-1). Severe reactions were observed in all animals given undiluted vaccine and all given dilutions from 1:10 to 1:80, with the remaining animals undergoing mild or inapparent reactions. All control animals underwent severe reactions to challenge. It was concluded that 1 ml of a 1:100 vaccine dose was considered both safe and efficacious.
Tenesi 2015 [58]	Vaccinated cattle were followed for five months, and no reactors were observed, concluding that the Muguga cocktail vaccine was safe.
Turasha 2005 [77]	An immunisation trial in Kenya reported a total of 87 reactors in 4000 immunised cattle (2.2%) and 46 mortalities (1.1%). Calf mortality had previously been 20–40%, hence safety and efficacy of the vaccine in Kenyan field conditions were demonstrated.

**Table 6:** Shed and transmission studies. These studies examined or reported the persistence of vaccine strains in vaccinated cattle as well as possible transmission of vaccine strains to unvaccinated animals.

Study	Year of Study	Country	Sample Size	Description of Possible Transmission of MC Vaccine Components
Amzati et al., 2019 [81]	2015	DRC and Burundi	480	Muguga vaccine component alleles present in all AEZs, may be associated with unrestricted movement of cattle in the region
Chatanga et al., 2020 [82]	2018	Malawi	254	Possible spread of vaccine components into unvaccinated cattle
De Deken et al., 2007 [83]	2003–2004	Comoros	21 & 6	Vaccinated can shed Muguga and Kiambu 5
Mwega et al., 2015 [84]	2012	Tanzania	39	Some field samples genetically related to Muguga vaccine isolate
Geysen et al., 1999 [85]	1996–1997	Zambia	12 (isolates)	Possible tick transmission of MC vaccine components
Gwakisa et al., 2020 [86]		Tanzania	410	Detection of vaccine component in co-grazing unvaccinated, possible vaccine transmission
Kerario et al., 2019 [87]	2014–2015	Tanzania	130	Indication of genotype sharing as Muguga vaccine isolate clustering despite no vaccination
Magulu et al., 2019 [88]		Tanzania	336	Muguga vaccine-specific alleles detected in unvaccinated cattle—potentially transmitted by ticks from vaccinated to unvaccinated
Mbyuzi et al., 2013 [80]	2010–2011	Tanzania	768	84% ECF cases in calves born to immunised cattle—possibility of vertical transmission of Muguga cocktail vaccine parasites
Nambota et al., 1997 [89]		Zambia	17 (stocks)	Possible introduction of *Theileria* stocks via MC use
Oura et al., 2004 [66]				Observed persistence of the Kiambu 5 strain in cattle for up to two years in Uganda, in comparison to the Muguga and Serengeti strains which were largely eliminated by three months. There was no evidence of transmission of vaccine stocks to unvaccinated in-contact animals (over a one-year period)
Oura et al., 2007 [90]	2000–2004	Uganda	36	Presence of Kiambu 5 stock in unvaccinated cattle, indicating “foreign” vaccine stocks will be introduced to local cattle and tick populations
Rukambile et al., 2014 [91]		Tanzania	102	Isolation of Muguga stock in areas where no immunisation. Cannot exclude possibility of vaccine derived as extensive nomadism, thus sharing of stocks likely to occur

## Data Availability

The data extraction files present all study details and can be found in Supplementary Files S3 and S4. Several studies are not publicly available, e.g., unpublished reports, but can be requested from the authors.

## Supplementary Materials

The following are available online at https://www.mdpi.com/article/10.3390/vaccines9111318/s1, Supplementary File S1: SYRCLE protocol; Supplementary File S2: PRISMA checklist; Supplementary File S3: Data extraction Excel file; Supplementary File S4: Data extraction Word file; Supplementary File S5: RoB mortality; Supplementary File S6: RoB severe reactions.
