# Supplementary material for: Safety and Efficacy of the East Coast Fever Muguga Cocktail Vaccine: A Systematic Review
Source: Vaccines (Basel). 2021 Nov 12;9(11):1318. doi: 10.3390/vaccines9111318 (PMC8623010; doi:10.3390/vaccines9111318)
Supplement: Supplementary file 1 [file vaccines-09-01318-s001.zip › Supplementary File S1 SYRCLE protocol.pdf]

Supplementary File S1: SYRCLE protocol.

SYSTEMATIC REVIEW PROTOCOL FOR ANIMAL INTERVENTION STUDIES

FORMAT BY SYRCLE (WWW.SYRCLE.NL)
VERSION 2.0 (DECEMBER 2014)

Item #	Section/Subsection/Item	Description	Check for approval
	**A. General**		
1.	Title of the review	Safety and efficacy of the East Coast fever Muguga cocktail vaccine: a systematic review	
2.	Authors (names, affiliations, contributions)	Dr Fiona K Allan[1] and Professor Andrew R Peters[1]<br>[1]Supporting Evidence Based Interventions in Livestock (SEBI-Livestock), The Royal (Dick) School of Veterinary Studies, The University of Edinburgh, Easter Bush Campus, Midlothian, EH25 9RG, UK	
3.	Other contributors (names, affiliations, contributions)	-	
4.	Contact person + e-mail address	Dr Fiona K Allan: fionakallan@gmail.com	
5.	Funding sources/sponsors	SEBI is funded by the Bill and Melinda Gates Foundation	
6.	Conflicts of interest	No conflicts of interest	
7.	Date and location of protocol registration	-	
8.	Registration number (if applicable)	-	
9.	Stage of review at time of registration	Preliminary searches completed<br>Piloting of study selection process completed<br>Formal screening of search results against eligibility criteria started	
	**B. Objectives**		
	Background		
10.	What is already known about this disease/model/intervention? Why is it important to do this review?	East Coast fever (ECF) in cattle is caused by the Apicomplexan protozoan parasite *Theileria parva*, and transmitted by the three-host tick *Rhipicephalus appendiculatus*, in Eastern, Central and Southern Africa. ECF is often fatal in cattle, with mortality as high as 90% in susceptible cattle. A vaccine, the Infection and Treatment Method (ITM), involves administration of three parasite isolates - the 'Muguga Cocktail' - and simultaneous treatment with oxytetracycline (OTC). Due to the unique nature of the vaccine, there were no established, dedicated clinical studies designed to assess its safety or efficacy during its development in the 1970s and it was not formally registered but was given special sanction for use in respective countries, so this systematic review aims to assess both the safety and efficacy. Although a disease of cattle, the impact of this disease to human lives can be great, as cattle support the livelihoods of smallholder farmers in countries where the diseases is often endemic.	

		The safety and efficacy of the Muguga Cocktail vaccine is potentially very important in relation to the vaccine as an intervention, supporting cattle health and productivity, and thus inextricably linked to the livelihood, well-being and health of farmers - exemplifying the 'One Health' concept.	
	Research question		
11.	Specify the disease/health problem of interest	East Coast fever	
12.	Specify the population/species studied	Cattle	
13.	Specify the intervention/exposure	ECF Muguga cocktail vaccine	
14.	Specify the control population	Non-vaccinated cattle	
15.	Specify the outcome measures	Efficacy of Muguga cocktail vaccination – (demonstrated as reduction in mortality and/or severe reactors in vaccinated compared to unvaccinated controls) and safety of Muguga cocktail (demonstrated by lack of local or systemic reactions in animals or the environment (including non-target animals)	
16.	State your research question (based on items 11-15)	What is the efficacy and safety of the Muguga cocktail vaccine?	
	**C. Methods**		
	Search and study identification		
17.	Identify literature databases to search (*e.g.* Pubmed, Embase, Web of science)	X MEDLINE via PubMed X Web of Science<br>☐SCOPUS ☐EMBASE<br>X Other, namely: CAB Direct, African Journals Online, and Google Scholar<br>☐Specific journal(s), namely:	
18.	Define electronic search strategies (*e.g.* use the step by step search guide[15] and animal search filters[20, 21])	When available, please add a supplementary file containing your search strategy: [will be included in manuscript]	
19.	Identify other sources for study identification	X Reference lists of included studies X Books<br>X Reference lists of relevant reviews<br>X Conference proceedings, namely:<br>X Contacting authors/ organisations, namely: where any data is confusing, attempts to contact authors will be made, in attempt to be able to use the study (those with unclear data will be excluded)<br>X Other, namely: theses and reports	
20.	Define search strategy for these other sources	Screening the sources for relevant titles and screening the abstracts of relevant titles	
	Study selection		
21.	Define screening phases (*e.g.* pre-screening based on title/abstract, full text screening, both)	1) Screening based on title and abstract<br>2) Full-text screening of eligible articles	
22.	Specify (a) the number of reviewers per screening phase and (b) how discrepancies will be resolved	Each phase: 1 observer (FA) will screen all articles, with a second observer (AP) reviewing a random sample to ensure cohesion, and differences will be resolved through discussion or by consulting a third reviewer	
	Define all inclusion and exclusion criteria based on:		

23.	Type of study (design)	Inclusion criteria: All studies describing Muguga cocktail use<br>Exclusion criteria: None	
24.	Type of animals/population (*e.g.* age, gender, disease model)	Inclusion criteria: cattle of all breeds, sex and age<br>Exclusion criteria: non-controlled studies	
25.	Type of intervention (*e.g.* dosage, timing, frequency)	Inclusion criteria: vaccination with Muguga cocktail. All dosages<br>Exclusion criteria: vaccination with alternative ECF vaccines	
26.	Outcome measures	Inclusion criteria: mortality rate of vaccinated and non-vaccinated cattle (where cause of mortality confirmed as ECF), severe reactions to challenge in vaccinated and non-vaccinated (using published classification gradation), seroconversion, onset and duration of immunity, vaccine efficacy (as per defined calculation)<br>Exclusion criteria: no relevant outcome measure	
27.	Language restrictions	Inclusion criteria: English<br>Exclusion criteria: all other non-English languages	
28.	Publication date restrictions	Inclusion criteria: all publication dates<br>Exclusion criteria: none	
29.	Other	NA	
30.	Sort and prioritize your exclusion criteria per selection phase	Selection phase:<br>1. Abstract only<br>2. Duplicated data<br>3. No controls<br>4. co-intervention e.g. inoculation with more than the three Muguga cocktail strains<br>5. Co-morbidity (ECF not confirmed cause of death)	
	Study characteristics to be extracted (for assessment of external validity, reporting quality)		
31.	Study ID (*e.g.* authors, year)	Author, title, year	
32.	Study design characteristics (*e.g.* experimental groups, number of animals)	Number of animals in experimental and control groups (sample sizes), study type (immunization trial, longitudinal etc), country of study, production system, agroecological zone	
33.	Animal model characteristics (*e.g.* species, gender, disease induction)	Cattle breed, age, weight and sex	
34.	Intervention characteristics (*e.g.* intervention, timing, duration)	Date of intervention, dosage of vaccination and dosage of oxytetracycline (OTC)	
35.	Outcome measures	ECF mortality, reported % vaccine efficacy, seroconversion, immunity onset, immunity duration, confirmation of ECF method (microscopy, post-mortem), safety (reactors or shed into non-target animals)	
36.	Other (*e.g.* drop-outs)	Excluded animals	
	Assessment risk of bias (internal validity) or study quality		
37.	Specify (a) the number of reviewers assessing the risk of bias/study quality in each study and (b) how discrepancies will be resolved	a) 2 reviewers, using predefined assessment criteria<br>b) discrepancies will be resolved by discussion	

38.	Define criteria to assess (a) the internal validity of included studies (*e.g.* selection, performance, detection and attrition bias) and/or (b) other study quality measures (*e.g.* reporting quality, power)	X By use of SYRCLE's Risk of Bias tool[4]<br>☐By use of SYRCLE's Risk of Bias tool, adapted as follows:<br>☐By use of CAMARADES' study quality checklist, e.g [22]<br>☐By use of CAMARADES' study quality checklist, adapted as follows:<br>☐Other criteria, namely:	
	Collection of outcome data		
39.	For each outcome measure, define the type of data to be extracted (*e.g.* continuous/dichotomous, unit of measurement)	ECF mortality: dichotomous (death or survival)<br>Reported % vaccine efficacy: continuous, unit: %<br>Seroconversion: %<br>Immunity onset: days<br>Immunity duration: days<br>ECF diagnosis: microscopy or post-mortem description<br>Safety (reactions): categorical<br>Safety (shed to non-target animals): continuous	
40.	Methods for data extraction/retrieval (*e.g.* first extraction from graphs using a digital screen ruler, then contacting authors)	Extraction from text and tables<br>Contacting authors by email where any confusion	
41.	Specify (a) the number of reviewers extracting data and (b) how discrepancies will be resolved	a) One reviewer (FA) will extract all data<br>b) Discrepancies will be resolved by discussion	
	Data analysis/synthesis		
42.	Specify (per outcome measure) how you are planning to combine/compare the data (*e.g.* descriptive summary, meta-analysis)	Meta-analysis of 'ECF mortality' and 'severe reactions', and descriptive synthesis of parameters '% vaccine efficacy', 'seroconversion', 'immunity onset', 'immunity duration', 'safety of vaccination' and 'safety – shed and spread of vaccine in non-target animals'. Additionally descriptive synthesis of 'safety and efficacy of OTC'	
43.	Specify (per outcome measure) how it will be decided whether a meta-analysis will be performed	Adequate quantitative data presented in studies i.e. mortality rates in intervention and control groups, and severe reactions in intervention and control groups. Ideally >10 studies per meta-analysis	
	If a meta-analysis seems feasible/sensible, specify (for each outcome measure):		
44.	The effect measure to be used (*e.g.* mean difference, standardized mean difference, risk ratio, odds ratio)	Odds ratio	
45.	The statistical model of analysis (*e.g.* random or fixed effects model)	Fixed effects model and Random effects, depending on assumed heterogeneity	
46.	The statistical methods to assess heterogeneity (*e.g.* I^2, Q)	I^2	
47.	Which study characteristics will be examined as potential source of heterogeneity (subgroup analysis)	Cattle breed<br>Challenge stabilate (known or natural field strains)<br>Vaccine batch (FAO1 vs FAO2)<br>Vaccine dose<br>Oxytetracycline concentration (20% vs 30%)<br>Time of year of study	

48.	Any sensitivity analyses you propose to perform	Sensitivity analyses will be conducted where there are 'outlying' outcome effects in individual studies, to assess how analysis of overall outcome effect may be affected	
49.	Other details meta-analysis (*e.g.* correction for multiple testing, correction for multiple use of control group)	Not planned	
50.	The method for assessment of publication bias	Critical visual assessment of Funnel plot	
Final approval by (names, affiliations):		Dr Fiona K Allan<br>Professor Andrew R Peters<br>Both affiliations - Supporting Evidence Based Interventions in Livestock (SEBI-Livestock), The Royal (Dick) School of Veterinary Studies, University of Edinburgh	Date: 10-05-2021
