# Supplementary material for: Safety and Efficacy of the East Coast Fever Muguga Cocktail Vaccine: A Systematic Review
Source: Vaccines (Basel). 2021 Nov 12;9(11):1318. doi: 10.3390/vaccines9111318 (PMC8623010; doi:10.3390/vaccines9111318)
Supplement: Supplementary file 1 [file vaccines-09-01318-s001.zip › Supplementary File S4 Data extraction.pdf]

Supplementary File S4: Data extraction.

**Reference**	**Study/Trial**	**ECF deaths in vaccinated**	**ECF deaths in unvaccinated**	**ECF diagnosis**	**Other study results**	**Inapparent reaction**	**Mild reactions**	**Moderate reactions**	**Severe reactions**	**ECF cases in vaccinated**	**ECF cases in unvaccinated**
**Mortality**											
**Dolan 1980 (separated into Dolan a, b, c & d)**	Challenge 1	0/5	3/4	Microscopy and LN biopsy		1/5 vacc (challenge with Idobogo stabilate 89)	4/5 vacc (challenge with Idobogo stabilate 89)		4/4 controls (challenge with Idobogo stabilate 89)		
	Challenge 2	0/5	3/4			3/5 vacc (challenge with Idobogo II stabilate 90)	2/5 vacc (challenge with Idobogo II stabilate 90)		4/4 controls		
	Challenge 3	0/4	3/5			3/4 vacc (challenge with Pugu I stabilate 119)	1/4 vacc (challenge with Pugu I stabilate 119)		5/5 controls		
	Challenge 4	0/5	4/5		Overall 0 vacc died from ECF compared to 72% of controls						
**Lynen 1999**	1	0/28	2/5	Blood smears and LN biopsies	14/28 got 30% OTC and 14/28 got 20% OTC		21.4% with 20% reduced to 7.1% with 30%				
	2				52/104 got 30% and 52/104 got 20% OTC				44% with 20% reduced to 7.4% with 30%		
**Lynen 2005**	Longitudinal	0/50	20/50	Post-mortem							
	Household survey	1%	37%								
**Martins 2010**		1/50	5/50	Blood smears and LN biopsies	97% vaccine efficacy in Endulen, NCA. 6% vacc reacted to vaccination					1/50	24/50

Reference	Study/Trial	ECF deaths in vaccinated	ECF deaths in unvaccinated	ECF diagnosis	Other study results	Inapparent reaction	Mild reactions	Moderate reactions	Severe reactions	ECF cases in vaccinated	ECF cases in unvaccinated
**Melewas 1999**	Tanzania mainland reactions to vacc and ECF deaths first large table) – no controls so can't do meta-analysis				576/14,628 (3.9%) reactors to vacc and 4 died of anaphylaxis						
	Immunisation trial 2	3/20	2/12		Considered inconclusive						
	Immunisation trial 3	3/15	7/14		Non- or moderate reactors: 8/15 vacc and 7/14 unvaccinated				7/15 vacc and 7/14 unvaccinated		
**Minjauw 1998**		3/46	17/49	Blood smears and LN biopsies		20/20 vacc and 9/20 non-vacc	3/20 non-vacc		8/20 non-vacc		
**Musisi 1989**		0/19	9/15	Microscopy and post-mortem	>70% vacc had detectable schizonts b/w 12-19 days compared to 60% controls. Detectable schizonts in vacc at 20-27 days reduced to 31% and increased to 80% in controls. No schizonts detected in vacc >27d, but detectable in controls until end of trial (50d)						
**Musisi 1996**	Challenge trial	1/17	5/12	Microscopy and post-mortem	Mean prepatent period to schizont was 7.9 ± 1.2 in controls and 10 ± 1.2 in vacc. Duration of detecting schizont was 15.5 ± 2.3 in controls and 9.4 ± 4.1 in MC vacc	1 non/mild to moderate reaction in controls and 14 or 15 (unclear) non/mild to moderate in vacc			6/12 controls severe (5 fatal) and 3 (also states 2, unclear) of 17 vacc severe (1 fatal)		
**Mutugi 1991**	Immun trial 1				37/50 vacc seroconverted	37/50 vacc and 17/17 Ab-positive (not vacc) had inapparent reaction to challenge	9/50 vacc	3/50 moderate/severe			
	Homologous challenge 2					48/50 vacc					
	Field challenge 3	2/67	1/10	Microscopy and post-mortem		82% inapparent reactions (don't know which data these are and actual numbers, and no controls to compare	14.9% mild reactions	2% moderate reactions	5.9% severe reactions		
**Radley 1975c**	Trial 1a (Kiambu1 challenge)	0/5	5/5	Blood smears and LN biopsies	Cattle vacc with MC cocktail had mild or no	3/5 vacc	2/5 vacc		5/5 controls		

Reference	Study/Trial	ECF deaths in vaccinated	ECF deaths in unvaccinated	ECF diagnosis	Other study results	Inapparent reaction	Mild reactions	Moderate reactions	Severe reactions	ECF cases in vaccinated	ECF cases in unvaccinated
					reaction to challenge, but variable rx in those vacc with one or two strains only						
	Trial 1b (T. lawrencei challenge)					2/5 vacc	3/5 vacc		5/5 controls		
	Trial 2a (Entebbe1 challenge)	0/6	3/5		Cattle vacc with combination had mild or no reaction to challenge to all three challenges, but susceptible had severe rx and many died		6/6 vacc		5/5 controls		
	Trial 2b (Entebbe2 challenge)	0/6	5/5			1/6 vacc	5/6 vacc		5/5 controls		
	Trial 2c (Ukunda challenge)	0/5	5/5			1/5 vacc	4/5 vacc		5/5 controls		
**Radley 1979**	Expt 1 (T. lawrencei)	3/20	18/20	Blood smears and LN biopsies		2/20 vacc no reaction	8/20 vacc mild reaction		20/20 controls severe reaction and 10/20 vacc severe reaction		
	Expt 2 (buffalo paddock)	3/6	5/6				2/6 vacc mild reaction		6/6 controls severe reaction and 4/6 vacc severe reaction		

Reference	Study/Trial	ECF deaths in vaccinated	ECF deaths in unvaccinated	ECF diagnosis	Other study results	Inapparent reaction	Mild reactions	Moderate reactions	Severe reactions	ECF cases in vaccinated	ECF cases in unvaccinated
	Expt 3 (groups 3 and 5 only) (buffalo paddock)	3/5	5/5				1/5 vacc mild reaction		5/5 controls severe reaction and 4/5 vacc severe reaction		
**Radley 1985**	Field trial 1	0/20		Blood smears and LN biopsies and post-mortem	Cannot use – control data unclear – 0/10 then 7/7 controls died ECF						
	Field trial 2	0/19	6/10								
	Field trial 3	0/24			Cannot use – control data unclear – 7/12 then 5 controls died ECF						
	Field trial 4	3/15	6/8								
**Schreuder 1976** (*also presented in Uilenberg 1978*)		1/9	9/9	Serology and microscopy							
**Sitt 2015**		9/12	9/12	Post-mortem, microscopy and serology	No significant difference in survival, disease onset or progression in vacc vs unvacc. All but one vacc seroconverted by day 35 (21-25days) cf all controls which remained negative by ELISA						

Reference	Study/Trial	ECF deaths in vaccinated	ECF deaths in unvaccinated	ECF diagnosis	Other study results	Inapparent reaction	Mild reactions	Moderate reactions	Severe reactions	ECF cases in vaccinated	ECF cases in unvaccinated
**Tenesi 2015**		0/123	7/119	Blood smears and LN biopsies	No reactors. Vacc efficacy 89.4%. 93% seroconversion					2/123 And in follow-up period, 35-150 days, 2 ECF cases (none died)	18/119 And in follow-up period 35-150 days, 8 ECF cases (7 died)
**Uilenberg 1976**		0/7	5/5	Serology, blood smears and LN biopsies	All vacc became infected with T. parva with no symptoms						
**Uilenberg 1977** *(also presented in Uilenberg 1978, trial 3)*		0/50	19/19	Blood smears and LN biopsies							
**Uilenberg 1978**	Field trial 1	0/7	7/7	Serology, blood smears and LN biopsies	This is the only unique dataset – others reported elsewhere						
	Field trial 2	1/9	9/9		Same data as Schreuder 1976						
	Field trial 3				Same data as Uilenberg 1977						
**% Efficacy**											
**Kiraithe 2015**				Blood smears and LN biopsies	97.8% efficacy in Ole Naishu and 78.4% in Mutara - buffalo interaction here may have reduced efficacy. Most vaccinated seroconverted by day 35. Mutara seroconversion 69% in vacc and 6.2% in controls; 83% Ole Naishu vacc and 3.1% in controls					Mutara: 4/65 Ole Naishu: 2/65	Mutara: 17/65 Ole Naishu: 54/65
**Lynen 2012**				Clinical signs and laboratory	97.6% efficacy in preventing ECF cases and 97.9% in preventing ECF deaths						
**Magwisha 2011**				Serology	70% efficacy (proportion +ve animals to total number animals) i.e. 835/1216 (70%) had high antibody titres >20PP						
**Martins 2010 (AGAIN)**		1/50	5/50	Blood smears and LN biopsies	97% vaccine efficacy. 6% vacc reacted to vaccination					1/50	24/50
**Nsubuga-Mutaka 1999**				Blood smears and serology	86% protection. 0.16% mortality in vacc		91% vacc mild-moderate reaction				
**Nsubuga-Mutaka 2000**	1990-1993				80% protection. 94% **seroconversion.** 90% **safety**						

Reference	Study/Trial	ECF deaths in vaccinated	ECF deaths in unvaccinated	ECF diagnosis	Other study results	Inapparent reaction	Mild reactions	Moderate reactions	Severe reactions	ECF cases in vaccinated	ECF cases in unvaccinated
	1994-1998				86% seroconversion and 90% **safety**						
**Tenesi 2015 (AGAIN)**		0/123	7/119	Blood smears and LN biopsies	No reactors. Vacc efficacy 89.4%. 93% seroconversion.					2/123 And in follow-up period, 35-150 days, 2 ECF cases (none died)	18/119 And in follow-up period 35-150 days, 8 ECF cases (7 died)
**Seroconversion**											
**Anon 1998**				Blood smears and LN biopsies	High proportion of seroconversion by 30 days (93.9% given 1:80 dose, and 84% given 1:100 dose). No reactions with either dose – both considered safe. Preferential use of the 30 mg/kg OTC						
**Anon 1999a**				Blood smears and LN biopsies	95% seroconversion with FAO2 and 100% seroconversion with FAO1 by **day 45 (immunity onset)** in both groups. 30mg/kg OTC		Only 1/31 with FAO1. No other reactors for either FAO1 or FAO2				

Reference	Study/Trial	ECF deaths in vaccinated	ECF deaths in unvaccinated	ECF diagnosis	Other study results	Inapparent reaction	Mild reactions	Moderate reactions	Severe reactions	ECF cases in vaccinated	ECF cases in unvaccinated
					preferable in reducing reactions						
**Atuhaire 2020a**				Blood smears and LN biopsies	35/41 vacc seroconverted (85.4%). Vaccine efficacy calculated as (32/40)-(6/41) ÷ (32/40) = 81.7%	25/41 vacc and 5/41 unvaccinated			6/41 vacc and 32/40 unvaccinated		
**Kazungu 2015**				Serology	99.2% sero**prevalence** in vaccinated and 96% sero**prevalence** in unvaccinated						
**Kiraithe 2015 (AGAIN)**				Blood smears and LN biopsies	97.8% efficacy in Ole Naishu and 78.4% in Mutara - buffalo interaction here may have reduced efficacy. Most vaccinated seroconverted by day 35. Mutara seroconversion 69% in vacc and 6.2% in controls; 83% Ole Naishu vacc and 3.1% in controls					Mutara: 4/65 Ole Naishu: 2/65	Mutara: 17/65 Ole Naishu: 54/65

Reference	Study/Trial	ECF deaths in vaccinated	ECF deaths in unvaccinated	ECF diagnosis	Other study results	Inapparent reaction	Mild reactions	Moderate reactions	Severe reactions	ECF cases in vaccinated	ECF cases in unvaccinated
**Mbassa 1998**				Blood smears and LN biopsies	38 of those immunized with -70C for 6 months were tested and had seroconverted by day 30. i.e. of those tested, 100% seroconversion by day 30. 1 calf given -70C for 6 weeks died from ECF. Other calf given -70 for 6 weeks, plus both calves given -70C for 6 months recovered without any treatment				1 severe in calf vacc (with -70 for 6 months) and died from disease other than ECF after monitoring period (day 24)		
**Patel 2019**				Blood smears and clinical signs	75-87% (mean 82%) seroconversion (in 62 vacc) [but no controls, but 2 ECF cases in non-vacc animals]		4/5 vacc (group 1) and 4/5 vacc (group 2)	1/5 group 2 vacc	2/2 controls and 1/10 vacc (group 1)		2 non-vacc
**Turasha 2011**				Blood and LN smears	Seroconversion after vacc was estimated at 73% (55.6-79.4% range, and mean 69.62%).						

Reference	Study/Trial	ECF deaths in vaccinated	ECF deaths in unvaccinated	ECF diagnosis	Other study results	Inapparent reaction	Mild reactions	Moderate reactions	Severe reactions	ECF cases in vaccinated	ECF cases in unvaccinated
					Pre-vacc 19.92 seroprev compared to 69.62 post-vacc seroprev i.e. good mean seroconversion. 17/3,017 vaccinated reacted (0.5%) and were treated for ECF and 3 (0.1%) died. Severity of reactors was not recorded and cause of deaths not certain						
**Wesonga 2013**				Blood and LN smears	Mutara and Old Naishu used MC vacc. Ol Pejeta uses 'natural immunisation'. In Mutara, pre-vacc was 3% and post-vacc seropos was 69%. In Ole Naishu, 1.6% pre-vac and 83% post-vacc. In Ol pejeta 2.9% pre-vacc and 51% post-vacc. For controls:					0/66 vacc at Mutara and 0/65 vacc at Ole Naishu. 4 vacc at Ol Pejeta but not sure out of how many (100?) and no controls as all treated the same	3/65 controls at Mutara and 0/66 controls at Ole Naishu

Reference	Study/Trial	ECF deaths in vaccinated	ECF deaths in unvaccinated	ECF diagnosis	Other study results	Inapparent reaction	Mild reactions	Moderate reactions	Severe reactions	ECF cases in vaccinated	ECF cases in unvaccinated
					In Mutura, 4.7% seropos at day 0 then 6.3% at day 35 In Ole Naishu, 3.1% day 0 then 3.1% at day 35						
**Severe reactions**											
**Atuhaire 2020a (AGAIN)**				Blood smears and LN biopsies	35/41 vacc seroconverted (85.4%). Vaccine efficacy calculated as (32/40)-(6/41) ÷ (32/40) = 81.7%	25/41 vacc and 5/41 unvaccinated			6/41 vacc and 32/40 unvaccinated		
**Bishop 2015**				Blood and LN smears	27 FAO1 immun and 27 controls	5/27 vacc and 6/27 controls	5/27 vacc and 7/27 controls	1/27 vacc	14/27 vacc and 14/27 controls		
**Dolan 1980 (separated into Dolan a, b, c & d) (AGAIN)**	Challenge 1	0/5	3/4	Microscopy and LN biopsy		1/5 vacc (challenge with Idobogo stabilate 89)	4/5 vacc (challenge with Idobogo stabilate 89)		4/4 controls (challenge with Idobogo stabilate 89)		
	Challenge 2	0/5	3/4			3/5 vacc (challenge with Idobogo II stabilate 90)	2/5 vacc (challenge with Idobogo II stabilate 90)		4/4 controls		
	Challenge 3	0/4	3/5			3/4 vacc (challenge with Pugu I stabilate 119)	1/4 vacc (challenge with Pugu I stabilate 119)		5/5 controls		
	Challenge 4	0/5	4/5		Overall 0 vacc died from ECF compared to 72% of controls						
**Melewas 1999 (AGAIN)**	Tanzania mainland reactions to				576/14,628 (3.9%) reactors to vacc and 4						

Reference	Study/Trial	ECF deaths in vaccinated	ECF deaths in unvaccinated	ECF diagnosis	Other study results	Inapparent reaction	Mild reactions	Moderate reactions	Severe reactions	ECF cases in vaccinated	ECF cases in unvaccinated
	vacc and ECF deaths first large table) – no controls so can't do meta-analysis				died of anaphylaxis						
	Immunisation trial 2	3/20	2/12		Considered inconclusive						
	Immunisation trial 3	3/15	7/14		Non- or moderate reactors: 8/15 vacc and 7/14 unvaccinated				7/15 vacc and 7/14 unvaccinated		
**Minjauw 1998 (AGAIN)**		3/46	17/49	Blood smears and LN biopsies		20/20 vacc and 9/20 non-vacc	3/20 non-vacc		8/20 non-vacc		
**Musisi 1996 (AGAIN)**	Challenge trial	1/17	5/12	Microscopy and post-mortem	Mean prepatent period to schizont was 7.9 ± 1.2 in controls and 10 ± 1.2 in vacc. Duration of detecting schizont was 15.5 ± 2.3 in controls and 9.4 ± 4.1 in MC vacc	1 non/mild to moderate reaction in controls and 14 or 15 (unclear) non/mild to moderate in vacc			6/12 controls severe (5 fatal) and 3 (also states 2, unclear) of 17 vacc severe (1 fatal)		
**Patel 2019 (AGAIN)**				Blood smears and clinical signs	75-87% (mean 82%) seroconversion (in 62 vacc) [but no controls, but 2 ECF cases in non-vacc animals]		4/5 vacc (group 1) and 4/5 vacc (group 2)	1/5 group 2 vacc	2/2 controls and 1/10 vacc (group 1)		2 non-vacc

Reference	Study/Trial	ECF deaths in vaccinated	ECF deaths in unvaccinated	ECF diagnosis	Other study results	Inapparent reaction	Mild reactions	Moderate reactions	Severe reactions	ECF cases in vaccinated	ECF cases in unvaccinated
**Radley 1975c (AGAIN)**	Trial 1a (Kiambu1 challenge)	0/5	5/5	Blood smears and LN biopsies	Cattle vacc with MC cocktail had mild or no reaction to challenge, but variable rx in those vacc with one or two strains only	3/5 vacc	2/5 vacc		5/5 controls		
	Trial 1b (T. lawrencei challenge)					2/5 vacc	3/5 vacc		5/5 controls		
	Trial 2a (Entebbe1 challenge)	0/6	3/5		Cattle vacc with combination had mild or no reaction to challenge to all three challenges, but susceptible had severe rx and many died		6/6 vacc		5/5 controls		
	Trial 2b (Entebbe2 challenge)	0/6	5/5			1/6 vacc	5/6 vacc		5/5 controls		
	Trial 2c (Ukunda challenge)	0/5	5/5			1/5 vacc	4/5 vacc		5/5 controls		
**Radley 1979 (AGAIN)**	Expt 1 (T. lawrencei)	3/20	18/20	Blood smears and LN biopsies		2/20 vacc no reaction	8/20 vacc mild reaction		20/20 controls severe reaction and 10/20 vacc severe reaction		
	Expt 2 (buffalo paddock)	3/6	5/6				2/6 vacc mild reaction		6/6 controls severe reaction and 4/6 vacc severe reaction		
	Expt 3 (groups 3 and 5 only) (buffalo paddock)	3/5	5/5				1/5 vacc mild reaction		5/5 controls severe reaction and 4/5 vacc severe reaction		
**Onset of immunity**											
**Anon 1998 (AGAIN)**				Blood smears and LN biopsies	High proportion of seroconversion by 30 days (93.9% given 1:80 dose, and 84% given 1:100 dose). No reactions with either dose – both considered safe. Preferential use of the 30 mg/kg OTC						
**Anon 1999a (AGAIN)**				Blood smears and LN biopsies	95% and 100% Seroconversion by 45 days. Safety of both vaccs at 1:80 dilution with OTC 30mg/kg. 30mg/kg OTC preferable in reducing reactions						
**Anon 1999b**				Lymph smears and serology	Seroconversion between 28 and 35 days						

Reference	Study/Trial	ECF deaths in vaccinated	ECF deaths in unvaccinated	ECF diagnosis	Other study results	Inapparent reaction	Mild reactions	Moderate reactions	Severe reactions	ECF cases in vaccinated	ECF cases in unvaccinated
**Kiraithe 2015 (AGAIN)**				Blood smears and LN biopsies	97.8% efficacy in Ole Naishu and 78.4% in Mutara – buffalo interaction here may have reduced efficacy. Most vaccinated seroconverted by day 35. Mutara seroconversion 69% in vacc and 6.2% in controls; 83% Ole Naishu vacc and 3.1% in controls					Mutara: 4/65 Ole Naishu: 2/65	Mutara: 17/65 Ole Naishu: 54/65
**Oura 2004**				Serology and PCR	Seroconversion occurred by 48 days (in 13 of 15 calves, 87%). Muguga/ Serengeti stock present at d48 but not d87, although present in one calf at d241 and d303 [Muguga and Serengeti largely eliminated by 3 months] Kiambu5 persisted in vacc cattle at						
					d48 and d303 (62%). No evidence of transmission to unvacc cattle over a 1-year period						
**Sitt 2015**		9/12	9/12	Post-mortem, microscopy and serology	No significant difference in survival, disease onset or progression in vacc vs unvacc. All but one vacc seroconverted by day 35 (21-25days) cf all controls which remained negative by ELISA						
**Duration of immunity**											
**Oura 2004 (AGAIN)**				Serology and PCR	Seroconversion occurred by 48 days (in 13 of 15 calves, 87%). Muguga/ Serengeti stock present at d48 but not d87, although present in one calf at d241 and d303 [Muguga and Serengeti largely eliminated by 3 months]						

Reference	Study/Trial	ECF deaths in vaccinated	ECF deaths in unvaccinated	ECF diagnosis	Other study results	Inapparent reaction	Mild reactions	Moderate reactions	Severe reactions	ECF cases in vaccinated	ECF cases in unvaccinated
					Kiambu5 persisted in vacc cattle at d48 and d303 (62%). No evidence of transmission to unvacc cattle over a 1-year period						
**Efficacy vs buffalo**											
**Bishop 2015 (AGAIN)**				Blood and LN smears	27 FAO1 immun and 27 controls	5/27 vacc and 6/27 controls	5/27 vacc and 7/27 controls	1/27 vacc	14/27 vacc and 14/27 controls		
**Di Giulio 2009**					95% reduction in overall calf mortality in NCA since vaccination (intense wildlife area) and 95% reduced calf mortality at TZ-Kenya border areas						
**Kiraithe 2015 (AGAIN)**				Blood smears and LN biopsies	97.8% efficacy in Ole Naishu and 78.4% in Mutara – buffalo interaction here may have reduced efficacy. Most vaccinated seroconverted by day 35. Mutara seroconversion					Mutara: 4/65 Ole Naishu: 2/65	Mutara: 17/65 Ole Naishu: 54/65

Reference	Study/Trial	ECF deaths in vaccinated	ECF deaths in unvaccinated	ECF diagnosis	Other study results	Inapparent reaction	Mild reactions	Moderate reactions	Severe reactions	ECF cases in vaccinated	ECF cases in unvaccinated
					69% in vacc and 6.2% in controls; 83% Ole Naishu vacc and 3.1% in controls						
**Martins 2010 (AGAIN)**		1/50	5/50	Blood smears and LN biopsies	97% vaccine efficacy in Endulen, NCA. 6% vacc reacted to vaccination					1/50	24/50
**Obara 2015**				Clinical signs and microscopy	53/113 developed clinical and parasitological features typical with buffalo-derived T. parva. [Shared field samples with Bishop 2015 & Pelle 2011]						
**Pelle 2011**				LN biopsies	findings consistent with results of vacc studies, with evidence that immunity by ITM with a mixture of parasites is not always effective against challenge with buffalo-derived parasites						

Reference	Study/Trial	ECF deaths in vaccinated	ECF deaths in unvaccinated	ECF diagnosis	Other study results	Inapparent reaction	Mild reactions	Moderate reactions	Severe reactions	ECF cases in vaccinated	ECF cases in unvaccinated
**Radley 1979 (AGAIN)**	Expt 1 (T. lawrencei)	3/20	18/20	Blood smears and LN biopsies		2/20 vacc no reaction	8/20 vacc mild reaction		20/20 controls severe reaction and 10/20 vacc severe reaction		
	Expt 2 (buffalo paddock)	3/6	5/6				2/6 vacc mild reaction		6/6 controls severe reaction and 4/6 vacc severe reaction		
	Expt 3 (groups 3 and 5 only) (buffalo paddock)	3/5	5/5				1/5 vacc mild reaction		5/5 controls severe reaction and 4/5 vacc severe reaction		
**Sitt 2015 (AGAIN)**		9/12	9/12	Post-mortem, microscopy and serology	No significant difference in survival, disease onset or progression in vacc vs unvacc All but one vacc seroconverted by day 35 (21-25days) cf all controls which remained negative by ELISA						
**Turasha 2005**		46/4000 (1.1%)			Mortality reduced to 1.1% from 20-40%. Overall reactor rate is 87 calves						

Reference	Study/Trial	ECF deaths in vaccinated	ECF deaths in unvaccinated	ECF diagnosis	Other study results	Inapparent reaction	Mild reactions	Moderate reactions	Severe reactions	ECF cases in vaccinated	ECF cases in unvaccinated
					(2.2%). MC vacc found to be safe and protective. Protects where buffalo exist						
**Vaccine efficacy as a statement / qualitative**											
**Akoolo 2008**					solid resistance to challenge with a potentially lethal dose of vaccine batch FAO-1 after vaccination with FAO-1						
**Anon 2007**					very high degree of vaccine efficacy, based on a 90% reduction in ECF morbidity/ mortality in ECF endemic areas following vacc with batch FAO1 in TZ and vaccine FAO-1 was accepted as safe in Tanzania. 30mg/kg OTC preferable						
**Homewood 2006**					"highly significant impact on survival" following use of						

Reference	Study/Trial	ECF deaths in vaccinated	ECF deaths in unvaccinated	ECF diagnosis	Other study results	Inapparent reaction	Mild reactions	Moderate reactions	Severe reactions	ECF cases in vaccinated	ECF cases in unvaccinated
					the MC ITM vaccine. Probability of a vacc animal surviving to 54 months was 97%, cf to 71% for those unvacc, and reduced calf mortality due to ECF from >20% to ~ 2%.						
**Mutugi 1997**				Blood and LN smears and serology	Direct evidence of efficacy of 1:80 dose of FAO-1 vs experimental challenge. 1:80 dose safe in terms of survival and few reactions						
**Mutugi date unknown**		5 (0.01%)			90% reduction in morbidity/mortality in Tanzania. No safety issues re. pregnant, lactating, breed, age, sex. No reactions in calves			2,700 (7%) vacc	1,000 (2.7%) vacc		
**Patel 2016**	Stage 1 immunized			Blood smears, serology and PCR	Severe reactions observed in all animals given undiluted	1/5 (1:80)<br>1/5 (1:120)<br>1/5 (1:160)	5/5 (1:40)<br>4/5 (1:80)<br>4/5 (1:120)<br>4/5 (1:160)				

Reference	Study/Trial	ECF deaths in vaccinated	ECF deaths in unvaccinated	ECF diagnosis	Other study results	Inapparent reaction	Mild reactions	Moderate reactions	Severe reactions	ECF cases in vaccinated	ECF cases in unvaccinated
					vaccine and all given dilutions from 1:10 to 1:80, with remaining animals undergoing mild or inapparent reactions. All control animals severe reactions to challenge. Concluded that 1ml of a 1:100 vaccine dose considered both safe and efficacious						
	Stage 1 challenged					5/5 (1:40)<br>3/5 (1:80)<br>4/5 (1:120)<br>4/5 (1:160)	2/5 (1:80)<br>1/5 (1:120)		1/5 (1:160)		
	Stage 2 immunized					3/5 (1:80)<br>4/5 (1:100)<br>2/5 (1:120)<br>3/5 (1:140)	1/5 (1:80)<br>1/5 (1:100)<br>2/5 (1:120)<br>2/5 (1:140)	1/5 (1:120)	1/5 (1:80)		
	Stage 2 challenged					1/5 (1:80)<br>3/5 (1:120)<br>1/5 (1:140)	1/5 (1:80)<br>3/5 (1:100)<br>2/5 (1:120)<br>2/5 (1:140)		3/5 (1:80)<br>2/5 (1:100)<br>2/5 (1:140)		
	Stage 3 immunized					1/5 (1:80)<br>1/5 (1:100)<br>1/5 (1:120)	4/5 (1:100)<br>4/5 (1:120)<br>5/5 (1:140)	4/5 (1:80)			
	Stage 3 challenged					1/5 (1:100)<br>1/5 (1:120)	5/5 (1:80)<br>4/5 (1:100)<br>4/5 (1:120)				

Reference	Study/Trial	ECF deaths in vaccinated	ECF deaths in unvaccinated	ECF diagnosis	Other study results	Inapparent reaction	Mild reactions	Moderate reactions	Severe reactions	ECF cases in vaccinated	ECF cases in unvaccinated
						4/5 (1:140)			1/5 (1:140)		
**Radley 1975c (AGAIN)**	Trial 1a (Kiambu1 challenge)	0/5	5/5	Blood smears and LN biopsies	Cattle vacc with MC cocktail had mild or no reaction to challenge, but variable rx in those vacc with one or two strains only	3/5 vacc	2/5 vacc		5/5 controls		
	Trial 1b (T. lawrencei challenge)					2/5 vacc	3/5 vacc		5/5 controls		
	Trial 2a (Entebbe1 challenge)	0/6	3/5		Cattle vacc with combination had mild or no reaction to challenge to all three challenges, but susceptible had severe rx and many died		6/6 vacc		5/5 controls		
	Trial 2b (Entebbe2 challenge)	0/6	5/5			1/6 vacc	5/6 vacc		5/5 controls		
	Trial 2c (Ukunda challenge)	0/5	5/5			1/5 vacc	4/5 vacc		5/5 controls		
**Steinaa 2018**				Clinical signs	no substantive difference in protection, with no broader a CTL response when using the cocktail, suggesting limited antigenic						

Reference	Study/Trial	ECF deaths in vaccinated	ECF deaths in unvaccinated	ECF diagnosis	Other study results	Inapparent reaction	Mild reactions	Moderate reactions	Severe reactions	ECF cases in vaccinated	ECF cases in unvaccinated
					diversity in the cocktail						
Temperature and efficacy											
**Atuhaire 2020b**				Blood and LN smears	No reduction in efficacy when stored at -80C for <30 days						
**Mbassa 1998 (AGAIN)**				Blood smears and LN biopsies	38 of those immunized with -70C for 6 months were tested and had seroconverted by day 30. i.e. of those tested, 100% seroconversion by day 30. 1 calf given -70C for 6 weeks died from ECF. Other calf given -70 for 6 weeks, plus both calves given -70C for 6 months recovered without any treatment				1 severe in calf vacc (with -70 for 6 months) and died from disease other than ECF after monitoring period (day 24)		
**Shed & Spread studies**											
**Safety as a statement / qualitative**											
**Anon 2007 (AGAIN)**					very high degree of vaccine efficacy, based on a 90% reduction in ECF morbidity/						

Reference	Study/Trial	ECF deaths in vaccinated	ECF deaths in unvaccinated	ECF diagnosis	Other study results	Inapparent reaction	Mild reactions	Moderate reactions	Severe reactions	ECF cases in vaccinated	ECF cases in unvaccinated
					mortality in ECF endemic areas following vacc with batch FAO1 in TZ and vaccine FAO-1 was accepted as safe in Tanzania. 30mg/kg OTC preferable						
**Anon 1998 (AGAIN)**				Blood smears and LN biopsies	High proportion of seroconversion by 30 days (93.9% given 1:80 dose, and 84% given 1:100 dose). No reactions with either dose – both considered safe. Preferential use of the 30 mg/kg OTC						
**Anon 1999a (AGAIN)**				Blood smears and LN biopsies	95% and 100% Seroconversion by 45 days. Safety of both vaccs at 1:80 dilution with OTC 30mg/kg. 30mg/kg OTC preferable in reducing reactions						

Reference	Study/Trial	ECF deaths in vaccinated	ECF deaths in unvaccinated	ECF diagnosis	Other study results	Inapparent reaction	Mild reactions	Moderate reactions	Severe reactions	ECF cases in vaccinated	ECF cases in unvaccinated
**ILRI 1996**				Blood smears and serology	sero-conversion and protection against homologous challenge from 1:10 to 1:80 vacc. All doses safe i.e. standard 1:80 dilution safe at least at double concentration and probably higher						
**Mbyuzi 2013**	Longitudinal 1			Blood and LN smears	3/768 vacc (0.4%) clinical reaction within 2 weeks of vacc. 431/515 ECF cases in calves born to vacc cows (83.7%) compared to 59 ECF cases in calves born to non-vacc cows (11.5%). Reported need for further safety evaluation and verification						
**Mutugi 1997 (AGAIN)**				Blood and LN smears and serology	Direct evidence of efficacy of 1:80 dose of FAO-1 vs experimental challenge.						

Reference	Study/Trial	ECF deaths in vaccinated	ECF deaths in unvaccinated	ECF diagnosis	Other study results	Inapparent reaction	Mild reactions	Moderate reactions	Severe reactions	ECF cases in vaccinated	ECF cases in unvaccinated
					1:80 dose safe in terms of survival and few reactions						
**Mutugi date unknown (AGAIN)**		5 (0.01%)			90% reduction in morbidity/mortality in Tanzania. No safety issues re. pregnant, lactating, breed, age, sex. No reactions in calves			2,700 (7%) vacc	1,000 (2.7%) vacc		
**Patel 2016 (AGAIN)**	Stage 1 immunized			Blood smears, serology and PCR	Severe reactions observed in all animals given undiluted vaccine and all given dilutions from 1:10 to 1:80, with remaining animals undergoing mild or inapparent reactions. All control animals severe reactions to challenge. Concluded that 1ml of a 1:100 vaccine dose considered	1/5 (1:80)<br>1/5 (1:120)<br>1/5 (1:160)	5/5 (1:40)<br>4/5 (1:80)<br>4/5 (1:120)<br>4/5 (1:160)				

Reference	Study/Trial	ECF deaths in vaccinated	ECF deaths in unvaccinated	ECF diagnosis	Other study results	Inapparent reaction	Mild reactions	Moderate reactions	Severe reactions	ECF cases in vaccinated	ECF cases in unvaccinated
					both safe and efficacious						
	Stage 1 challenged					5/5 (1:40)<br>3/5 (1:80)<br>4/5 (1:120)<br>4/5 (1:160)	2/5 (1:80)<br>1/5 (1:120)		1/5 (1:160)		
	Stage 2 immunized					3/5 (1:80)<br>4/5 (1:100)<br>2/5 (1:120)<br>3/5 (1:140)	1/5 (1:80)<br>1/5 (1:100)<br>2/5 (1:120)<br>2/5 (1:140)	1/5 (1:120)	1/5 (1:80)		
	Stage 2 challenged					1/5 (1:80)<br>3/5 (1:120)<br>1/5 (1:140)	1/5 (1:80)<br>3/5 (1:100)<br>2/5 (1:120)<br>2/5 (1:140)		3/5 (1:80)<br>2/5 (1:100)<br>2/5 (1:140)		
	Stage 3 immunized					1/5 (1:80)<br>1/5 (1:100)<br>1/5 (1:120)	4/5 (1:100)<br>4/5 (1:120)<br>5/5 (1:140)	4/5 (1:80)			
	Stage 3 challenged					1/5 (1:100)<br>1/5 (1:120)<br>4/5 (1:140)	5/5 (1:80)<br>4/5 (1:100)<br>4/5 (1:120)		1/5 (1:140)		
**Turasha 2005 (AGAIN)**		46/4000 (1.1%)			Mortality reduced to 1.1% from 20-40%. Overall reactor rate is 87 calves (2.2%). MC vacc found to be safe and protective. Protects where buffalo exist						
**OTC safety and efficacy**											
**Anon 1998 (AGAIN)**				Blood smears and LN biopsies	High proportion of seroconversion by 30 days						

Reference	Study/Trial	ECF deaths in vaccinated	ECF deaths in unvaccinated	ECF diagnosis	Other study results	Inapparent reaction	Mild reactions	Moderate reactions	Severe reactions	ECF cases in vaccinated	ECF cases in unvaccinated
					(93.9% given 1:80 dose, and 84% given 1:100 dose). No reactions with either dose – both considered safe. Preferential use of the 30 mg/kg OTC						
**Anon 1999a (AGAIN)**				Blood smears and LN biopsies	95% and 100% Seroconversion by 45 days. Safety of both vaccs at 1:80 dilution with OTC 30mg/kg. 30mg/kg OTC preferable in reducing reactions						
**Anon 2007 (AGAIN)**					very high degree of vaccine efficacy, based on a 90% reduction in ECF morbidity/ mortality in ECF endemic areas following vacc with batch FAO1 in TZ and vaccine FAO-1 was accepted as safe in Tanzania.						

Reference	Study/Trial	ECF deaths in vaccinated	ECF deaths in unvaccinated	ECF diagnosis	Other study results	Inapparent reaction	Mild reactions	Moderate reactions	Severe reactions	ECF cases in vaccinated	ECF cases in unvaccinated
					30mg/kg OTC preferable						
**Clarke 1999**					Two OTC formulations (Merial and Boehringer) at 20mg/kg SC or IM were bioequivalent						
**Di Giulio 2000**				Blood smears and LN biopsies and serology	30mg/kg optimal dose for ECF-ITM, as 40mg/kg reduced reactions but also fewer seroconversion						
**Lynen 1999**	1	0/28	2/5	Blood smears and LN biopsies	14/28 got 30% OTC and 14/28 got 20% OTC		21.4% with 20% reduced to 7.1% with 30%				
