# Supplementary material for: Safety and Efficacy of the East Coast Fever Muguga Cocktail Vaccine: A Systematic Review
Source: Vaccines (Basel). 2021 Nov 12;9(11):1318. doi: 10.3390/vaccines9111318 (PMC8623010; doi:10.3390/vaccines9111318)
Supplement: Supplementary file 1 [file vaccines-09-01318-s001.zip › Supplementary File S5 RoB mortality.pdf]

Supplementary File S5: RoB mortality.

Risk of bias in studies included in the meta-analysis of mortality, using the Risk of Bias tool.

Study	1. Was the allocation sequence adequately generated and applied?	2. Were the groups similar at baseline or were they adjusted for confounders in the analysis?	3. Was the allocation to the different groups adequately concealed during assignment?	4. Were the animals randomly housed during the experiment?	5. Were the caregivers and/or investigators blinded from knowledge which intervention each animal received during the experiment?	6. Were animals selected at random for outcome assessment?	7. Was the outcome assessor blinded? *	8. Were incomplete outcome data adequately addressed?	9. Are reports of the study free of selective outcome reporting?	10. Was the study apparently free of other problems that could result in high risk of bias?	Summary on the overall risk of study bias
Dolan *et al.*, 1980 [1]	No	Yes	No	Unclear	No	Unclear	Yes	Yes	Yes	No	High
Lynen *et al.*, 1999 [2]	No	Yes	No	Unclear	No	Unclear	Yes	Yes	Yes	Yes	High
Lynen *et al.*, 2005 [3]	Yes	Yes	No	Unclear	No	Unclear	Yes	Yes	Yes	Yes	High
Martins *et al.*, 2010 [4]	Yes	Yes	No	Yes	No	Unclear	Yes	Yes	Yes	Yes	High
Melewas, Majaliwa and Lynen, 1999 [5]	No	Unclear	No	Unclear	No	Unclear	Yes	Yes	Yes	Yes	High
Minjauw *et al.*, 1998 [6]	Yes	Yes	No	Yes	No	Unclear	Yes	Yes	Yes	Yes	High
Musisi *et al.*, 1989 [7]	No	Yes	No	Unclear	No	Unclear	Yes	Yes	Yes	Yes	High
Musisi *et al.*, 1996 [8]	No	Yes	No	Unclear	No	Unclear	Yes	Yes	Yes	Yes	High
Mutugi and Otim, 1991 [9]	No	Unclear	No	Unclear	No	Unclear	Yes	No	Yes	Yes	High
Radley *et al.*, 1975c [10]	No	Unclear	No	Unclear	No	Unclear	Yes	Yes	Yes	Yes	High
Radley *et al.*, 1979 [11]	No	Unclear	No	Unclear	No	Unclear	Yes	Yes	Yes	Yes	High
Radley, 1985 [12]	No	Unclear	No	Unclear	No	Unclear	Yes	No	Yes	Yes	High
Schreuder *et al.*, 1976 [13]	No	Yes	No	Unclear	No	Unclear	Yes	Yes	Yes	Yes	High
Sitt *et al.*, 2015 [14]	Yes	Yes	Unclear	Yes	Yes	Unclear	Yes	Yes	Yes	Yes	Low
Tenesi, 2015 [15]	Yes	Yes	Unclear	Unclear	No	Unclear	Yes	Unclear	Yes	Yes	Moderate
Uilenberg *et al.*, 1976 [16]	No	Yes	No	Unclear	No	Unclear	Yes	Yes	Yes	Yes	High
Uilenberg *et al.*, 1977 [17]	No	Yes	No	Unclear	No	Unclear	Yes	Yes	Yes	Yes	High
Uilenberg *et al.*, 1978 [18]	No	Yes	No	Unclear	No	Unclear	Yes	Yes	Yes	Yes	High

Yes = low risk; No = high risk; Unclear = unclear risk. *Regarding signalling question 7, Outcome assessors were not blinded, but it was judged that the outcome – mortality - is unlikely influenced by lack of blinding, therefore low risk of bias.

References (as listed in Supplementary File 5) - **please note that some of these are also cited in the manuscript and are numbered differently**

1. Dolan, T.T.; Radley, D.E.; Brown, C.G.D.; Cunningham, M.P.; Morzaria, S.P.; Young, A.S. East Coast fever: 4. Further Studies on the Protection of Cattle Immunized with a Combination of Theilerial Strains. *Vet. Parasitol.* **1980**, *6*, 325–332.
2. Lynen, G.; Melewas, J.; Majaliwa, K.; Bakuname, C.; di Giulio, G. The Use of a 30% Formulation of Oxytetracycline Long-Acting in East Coast fever Immunisation in Tanzania. In Proceedings of the Animal Health and Production for Development. Proceedings of IX International Conference of Associations of Institutions of Tropical veterinary medicine, Harare, Zimbabwe 14-18 September 1998, vol. 1; Mukaratirwa, S., Obwolo, M.J., Eds.; University of Utrecht Press, 1999; pp. 367–375.
3. Lynen, G.; di Giulio, G.; Homewood, K.; Reid, R.; Mwilawa, A. Deployment of a Live ECF Vaccine in Pastoral Areas: Lessons Learned from Tanzania. In Proceedings of the The role of biotechnology in animal agriculture to address poverty in Africa: opportunities and challenges. Proceedings of the 4th All African Conference on Animal Agriculture and the 31st Annual Meeting of the Tanzania Society for Animal Production (TSAP; 2005; pp. 193–201.
4. Martins, S.B.; Di Giulio, G.; Lynen, G.; Peters, A.; Rushton, J. Assessing the impact of East Coast Fever immunisation by the infection and treatment method in Tanzanian pastoralist systems. *Prev. Vet. Med.* **2010**, *97*, 175–182, doi:10.1016/j.prevetmed.2010.09.018.
5. Melewas, J.; Majaliwa, M.; Lynen, L. ECF immunisation in Tanzania. In Proceedings of the FAO/ILRI/OAU-IBAR Workshop "Live Vaccines for Theileria Parva: Deployment in Eastern, Central and Southern Africa", held at ILRI, Nairobi, Kenya 10-12 March 1997; Morzaria, S., Williamson, S., Eds.; ILRI, 1999; pp. 13–20.
6. Minjauw, B.; Otte, M.J.; James, A.D.; De Castro, J.J.; Sinyangwe, P. Effect of different East Coast fever control strategies on disease incidence in traditionally managed Sanga cattle in Central Province of Zambia. *Prev. Vet. Med.* **1998**, *35*, 101–113, doi:10.1016/S0167-5877(98)00053-1.
7. Musisi, F.L.; Quiroga, J.C.; Ngulube, B.; Kanhai, G.K. An East Coast fever immunization field trial at Kasoba, Malawi. In Proceedings of the Theileriosis in eastern, central and southern Africa. Proceedings of a workshop on East Coast fever immunization held in Lilongwe, Malawi 20-22 September 1988; International Laboratory for Research on Animal Diseases, 1989; pp. 71–76.
8. Musisi, F.L.; Quiroga, J.C.; Kanhai, G.K.; Kamweno, S.P.; Mzoma, F.J.; Njuguna, L.M. Theileria parva (Kasoba) isolated and tested in cured cattle after infection with other stocks of Theileria parva. *Rev. Breed. Vet. Med. Trop. Ctries.* **1996**, *49*, 42–45, doi:10.19182/remvt.9544.
9. Mutugi, J.J.; Otim, C.P. East Coast fever immunization trials in Uganda. In Proceedings of the Ticks and tick-borne disease control: Proceedings of a joint OAU, FAO, and ILRAD workshop held at Kampala, Uganda, 12-14 September 1991; 1991; pp. 9–10.
10. Radley, D.E.; Brown, C.G.D.; Cunningham, M.P.; Kimber, C.D.; Musisi, F.L.; Payne, R.C.; Purnell, R.E.; Stagg, S.M.; Young, A.S. East coast fever: 3. Chemoprophylactic immunization of cattle using oxytetracycline and a combination of theilerial strains. *Vet. Parasitol.* **1975**, *1*, 51–60, doi:10.1016/0304-4017(75)90007-2.
11. Radley, D.E.; Young, A.S.; Grootenhuis, J.G.; Cunningham, M.P.; Dolan, T.T.; Morzaria, S.P. Further studies on the immunization of cattle against Theileria lawrencei by infection and chemoprophylaxis. *Vet. Parasitol.* **1979**, *5*, 117–128, doi:10.1016/0304-4017(79)90003-7.
12. Radley, D.E. East Coast fever immunization – field trials in Malawi. In Proceedings of the Immunization Against Theileriosis in Africa, ed. A.D. Irvin, held in Nairobi, Kenya, 1-5 October 1984; Irvin, A.D., Ed.; ILRAD: Nairobi, 1985; pp. 58–61.
13. Schreuder, B.E.C.; Silayo, R.S.; Uilenberg, G.; Mpangala, C.; Sanga, H.J.N. Studies on Theileriidae (Sporozoa) in Tanzania. VI. Second Field Trial on Immunization against Cattle Theileriosis. *Tropenmed. Parasitol.* **1976**, *27*, 26–34.
14. Sitt, T.; Poole, E.J.; Ndambuki, G.; Mwaura, S.; Njoroge, T.; Omondi, G.P.; Mutinda, M.; Mathenge, J.; Prettejohn, G.; Morrison, W.I.; et al. Exposure of vaccinated and naive cattle to natural challenge from buffalo-derived Theileria parva. *Int. J. Parasitol. Parasites Wildl.* **2015**, *4*, 244–251, doi:10.1016/j.ijppaw.2015.04.006.
15. Tenesi, M.G. An assessment of the efficacy of infection and treatment method against east coast fever and the financial and economic benefits in narok county of Kenya, University of Nairobi, 2015.
16. Uilenberg, G.; Schreuder, B.E.C.; Silayo, R.S.; Mpangala, C. Studies on Theileriidae (Sporozoa) in Tanzania. IV. A Field Trial on Immunization against East Coast Fever (Theileria parva Infection of Cattle). *Tropenmed. Parasitol.* **1976**, *27*, 329–336.
17. Uilenberg, G.; Silayo, R.S.; Mpangala, C.; Tondeur, W.; Tatchell, R.J.; Sanga, H.J.N. Studies on Theileridae (Sporozoa) in Tanzania. X. A Large-scale Field Trial on Immunization Against Cattle Theileriosis. *Tropenmed. Parasitol.* **1977**, *28*, 499–506.
18. Uilenberg, G.; Schreuder, B.E.C.; Mpangala, C.; Silayo, R.S.; Tondeur, W.; Tatchell, R.J.; Sanga, H.J.N. Immunization Against East Coast fever. In Proceedings of the Tick-borne diseases and their vectors: proceedings of an international conference held in Edinburgh from the 27th September to the 1st October, 1976; Wilde, J.K.H., Ed.; Edinburgh University Press, 1978; pp. 307–314.
