# Supplementary material for: Safety and Efficacy of the East Coast Fever Muguga Cocktail Vaccine: A Systematic Review
Source: Vaccines (Basel). 2021 Nov 12;9(11):1318. doi: 10.3390/vaccines9111318 (PMC8623010; doi:10.3390/vaccines9111318)
Supplement: Supplementary file 1 [file vaccines-09-01318-s001.zip › Supplementary File S6 RoB severe reactions final.pdf]

Supplementary File S6: RoB severe reactions final.

Risk of bias of studies included in the meta-analysis of efficacy in response to challenge (severe reactions), using the Risk of Bias tool.

Study	1. Was the allocation sequence adequately generated and applied?	2. Were the groups similar at baseline or were they adjusted for confounders in the analysis?	3. Was the allocation to the different groups adequately concealed during assignment?	4. Were the animals randomly housed during the experiment?	5. Were the caregivers and/or investigators blinded from knowledge which intervention each animal received during the experiment?	6. Were animals selected at random for outcome assessment?	7. Was the outcome assessor blinded?	8. Were incomplete outcome data adequately addressed?	9. Are reports of the study free of selective outcome reporting?	10. Was the study apparently free of other problems that could result in high risk of bias?	Summary on the overall risk of study bias
Atuhaire *et al.*, 2020 [1]	Yes	Yes	No	Unclear	No	Unclear	No	Yes	Yes	Yes	High
Bishop *et al.*, 2015 [2]	No	Yes	No	Unclear	No	Unclear	No	Yes	Yes	Yes	High
Dolan *et al.*, 1980 [3]	No	Yes	No	Unclear	No	Unclear	No	Yes	Yes	No	High
Melewas, Majaliwa and Lynen, 1999 [4]	No	Unclear	No	Unclear	No	Unclear	No	Yes	Yes	Yes	High
Minjauw *et al.*, 1998 [5]	Yes	Yes	No	Yes	No	Unclear	No	Yes	Yes	Yes	High
Musisi *et al.*, 1996 [6]	No	Yes	No	Unclear	No	Unclear	No	Yes	Yes	Yes	High
Patel *et al.*, 2019 [7]	Yes	Yes	No	Unclear	No	Unclear	No	Yes	Yes	Yes	High
Radley *et al.*, 1975c [8]	No	Unclear	No	Unclear	No	Unclear	No	Yes	Yes	Yes	High
Radley *et al.*, 1979 [9]	No	Unclear	No	Unclear	No	Unclear	No	Yes	Yes	Yes	High

Yes = low risk; No = high risk; Unclear = unclear risk.

References (as listed in Supplementary File 6) – **please note that some of these are also cited in the manuscript and are numbered differently**

1. Atuhaire, D.K.; Muleya, W.; Mbao, V.; Bazarusanga, T.; Gafarasi, I.; Salt, J.; Namangala, B.; Musoke, A.J. Sequence diversity of cytotoxic T cell antigens and satellite marker analysis of Theileria parva informs the immunization against East Coast fever in Rwanda. *Parasit. Vectors* **2020**, *13*, doi:10.1186/s13071-020-04322-9.
2. Bishop, R.; Hemmink, J.; Morrison, W.; Weir, W.; Toye, P.; Sitt, T.; Spooner, P.; Musoke, A.; Skilton, R.; Odongo, D. The African buffalo parasite Theileria. sp. (buffalo) can infect and immortalize cattle leukocytes and encodes divergent orthologues of Theileria parva antigen genes. *Int. J. Parasitol. Parasites Wildl.* **2015**, *4*, 333–342, doi:10.1016/j.ijppaw.2015.08.006.
3. Dolan, T.T.; Radley, D.E.; Brown, C.G.D.; Cunningham, M.P.; Morzaria, S.P.; Young, A.S. East Coast fever: 4. Further Studies on the Protection of Cattle Immunized with a Combination of Theilerial Strains. *Vet. Parasitol.* **1980**, *6*, 325–332.
4. Melewas, J.; Majaliwa, M.; Lynen, L. ECF immunisation in Tanzania. In Proceedings of the FAO/ILRI/OAU-IBAR Workshop "Live Vaccines for Theileria Parva: Deployment in Eastern, Central and Southern Africa", held at ILRI, Nairobi, Kenya 10-12 March 1997; Morzaria, S., Williamson, S., Eds.; ILRI, 1999; pp. 13–20.
5. Minjauw, B.; Otte, M.J.; James, A.D.; De Castro, J.J.; Sinyangwe, P. Effect of different East Coast fever control strategies on disease incidence in traditionally managed Sanga cattle in Central Province of Zambia. *Prev. Vet. Med.* **1998**, *35*, 101–113, doi:10.1016/S0167-5877(98)00053-1.
6. Musisi, F.L.; Quiroga, J.C.; Kanhai, G.K.; Kamweno, S.P.; Mzoma, F.J.; Njuguna, L.M. Theileria parva (Kasoba) isolated and tested in cured cattle after infection with other stocks of Theileria parva. *Rev. Breed. Vet. Med. Trop. Ctries.* **1996**, *49*, 42–45, doi:10.19182/remvt.9544.
7. Patel, E.; Mwaura, S.; Di Giulio, G.; Cook, E.A.J.; Lynen, G.; Toye, P. Infection and treatment method (ITM) vaccine against East Coast fever: reducing the number of doses per straw for use in smallholder dairy herds by thawing, diluting and refreezing already packaged vaccine. *BMC Vet. Res.* **2019**, *15*, doi:10.1186/s12917-019-1787-y.
8. Radley, D.E.; Brown, C.G.D.; Cunningham, M.P.; Kimber, C.D.; Musisi, F.L.; Payne, R.C.; Purnell, R.E.; Stagg, S.M.; Young, A.S. East coast fever: 3. Chemoprophylactic immunization of cattle using oxytetracycline and a combination of theilerial strains. *Vet. Parasitol.* **1975**, *1*, 51–60, doi:10.1016/0304-4017(75)90007-2.
9. Radley, D.E.; Young, A.S.; Grootenhuis, J.G.; Cunningham, M.P.; Dolan, T.T.; Morzaria, S.P. Further studies on the immunization of cattle against Theileria lawrencei by infection and chemoprophylaxis. *Vet. Parasitol.* **1979**, *5*, 117–128, doi:10.1016/0304-4017(79)90003-7.
